# Perception, assessment, and coaching: a systematic review and taxonomy of computer vision-based physical rehabilitation techniques

**DOI:** 10.3389/fresc.2026.1906327

**Published:** 2026-07-20

**Authors:** Ping Ye, Yu Li, Mengjian Qu, Jing Liu, Xiangqing Lu, Wenhui Cao, Rong Wang, Yaping Wan, Tao Zhu, Jun Zhou

**Affiliations:** 1 School of Computer Science, University of South China, Hengyang, China; 2 Department of Rehabilitation, The First Affiliated Hospital, Hengyang Medical School, University of South China, Hengyang, China; 3 Department of Rehabilitation Sciences, The Hong Kong Polytechnic University, Kowloon, Hong Kong SAR, China; 4 School of Nursing, University of South China, Hengyang, China

**Keywords:** action quality assessment, biomechanical digital twin, computer vision, generative AI, physical rehabilitation, tele-rehabilitation

## Abstract

The digital transformation of rehabilitation training has become a public health imperative driven by a global demand that outstrips professional medical resources and is compounded by a deficit in public rehabilitation literacy. As traditional hospital-centric models reach their scalability limits, there is a critical necessity for accessible home-based care solutions to ensure patients do not miss optimal recovery windows. To evaluate computer vision as a potential solution, this paper conducts a systematic review following the PRISMA 2020 reporting framework. We identify that its clinical migration faces a profound “Paradigm Gap” across three critical domains which this study aims to address: (1) the **Perception Domain**, where algorithms are constrained by inherent reconstruction ambiguities and pathological data scarcity; (2) the **Assessment Domain**, where a “semantic gap” persists between low-level features and clinical reasoning; and (3) the **Coaching Domain**, where feedback mechanisms fail to translate summative “Knowledge of Results (KR)” into actionable “Knowledge of Performance (KP).” We formulate and adopt “Perception, Assessment, and Coaching (PAC)” as a novel taxonomy to serve as a logical grid for systematically analyzing existing literature and elucidating technical pathways required to resolve these clinical challenges. This review synthesizes the technological landscape into three evolutionary trajectories: (1) In Perception, research is shifting toward constructing biomechanically consistent digital twins to eliminate visual hallucinations. (2) In Assessment, frontier methods are establishing interpretable clinical reasoning engines to achieve a leap from engineering parameters to Evidence-Based Medicine (EBM) evidence. (3) In Coaching, focus lies in precise movement correction via semantic translation and multimodal strategies to support motor relearning. Furthermore, we explore the potential of Generative AI and Multimodal Large Language Models (MLLMs) in reshaping interaction paradigms (e.g., Visual Self-Modeling) alongside critical discussions on ethical boundaries. Through a systematic literature review, this paper elucidates the task boundaries of rehabilitation vision and constructs the PAC taxonomy. It provides a robust theoretical roadmap and forward-looking guidance for the design of the next generation of clinically valid intelligent rehabilitation systems.

## 1 Introduction

### 1.1 Background

The profound transformation of global demographics, driven by an unprecedented wave of aging, is rapidly reshaping the public health landscape. Consequently, Rehabilitation Medicine has evolved from a supplementary service into a fundamental pillar of the global health system. As epidemiological characteristics shift, the medical community faces multifaceted challenges. On one hand, the aging society has significantly exacerbated the disease burden of neurodegenerative disorders such as stroke and Parkinson’s disease. On the other hand, the incidence of sports injuries [e.g., Anterior Cruciate Ligament (ACL) reconstruction] and chronic musculoskeletal disorders (e.g., Osteoarthritis) is surging across all age demographics (1).

Data from the Global Burden of Disease Study 2021 indicate that approximately 2.4 billion individuals worldwide are in urgent need of rehabilitation interventions (2, 3). However, confronted with this exponentially growing population suffering from generalized motor dysfunction, existing healthcare systems are constrained by a dual deficit of “Resources and Cognition” (4, 5). On the supply side, there is a severe, inelastic shortage of professional Physical Therapists (PTs), making the traditional “one-on-one” manual supervision model unsustainable. On the demand side, the public generally lacks scientific “Rehabilitation Literacy,” often conflating rehabilitation with simple mechanical repetition while overlooking the critical roles of neural control and movement quality. The superposition of resource scarcity and cognitive bias causes a vast number of patients to miss optimal recovery windows, confronting the traditional hospital-centric model with a severe “**Scalability Bottleneck**.”

### 1.2 Challenges and bottlenecks

To mitigate this public health crisis, the extension of rehabilitation scenarios from clinical settings to the home (Home-based Rehabilitation) has emerged as an inevitable trend. However, mainstream Telerehab solutions remain at a rudimentary level, primarily relying on “video calls” or passive “video watching.” This “supervision vacuum,” detached from professional oversight, renders patients highly susceptible to compensatory injuries caused by incorrect movements and leads to high attrition rates due to the monotony of training (6, 7). Confronted with this dilemma, there is an urgent need for intelligent rehabilitation systems capable of digitizing clinical expertise to achieve scalable and precise guidance.

**Figure 1:**
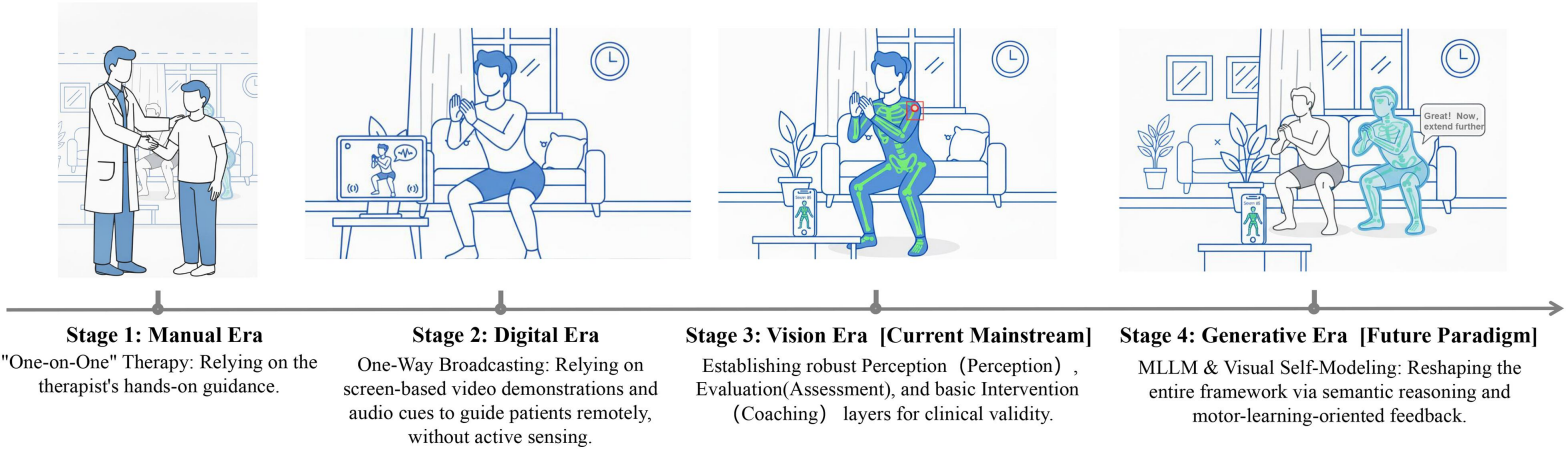
The technological evolution roadmap of rehabilitation training systems: A synthesis of the transition from manual supervision to the Generative AI-empowered **analytical model** based on “Perception, Assessment, and Coaching” (PAC). This diagram categorizes the paradigm shifts in rehabilitation computing literature across four distinct stages: **(1) Stage 1 (Manual Era):** Traditional one-on-one therapist intervention, inherently limited by labor intensity and scalability constraints documented in early research. **(2) Stage 2 (Digital Era):** Remote instruction relying on one-way video broadcasting without active sensing or automated feedback mechanisms. **(3) Stage 3 (Vision Era):** The current mainstream paradigm leveraging computer vision to establish core computational **domains** of Perception, Assessment, and Coaching to meet basic quantitative needs. **(4) Stage 4 (Generative Era):** The **emerging future landscape** driven by Multimodal Large Language Models (MLLMs) and Generative AI. By synthesizing semantic reasoning and **Visual Self-Modeling (VSM)**, this stage represents a shift from passive observation toward autonomous rehab agents capable of motor-learning-oriented interaction.

In recent years, driven by breakthroughs in deep learning, markerless pose estimation technology based on ubiquitous RGB video has provided a novel opportunity for automated home rehabilitation guidance, owing to its low cost and high accessibility. While recent review articles have extensively covered general Human Pose Estimation (HPE) architectures (8, 9) and multi-view reconstruction techniques (10), these works predominantly focus on engineering metrics such as mAP or MPJPE on standard datasets. Although some recent clinical benchmarking studies (11) have attempted to evaluate the angular accuracy of visual systems using Inertial Measurement Units (IMUs) as a reference, most existing research treats perception, assessment, and coaching as fragmented modules. There is a distinct lack of a unified framework that organically integrates biomechanical constraints with motor learning theories. It is precisely due to this misalignment between engineering objectives and clinical requirements that, despite significant progress in computer vision for general Human Action Recognition (HAR), migrating these technologies to rigorous medical rehabilitation scenarios still encounters a profound “Paradigm Gap.” The technological evolution of rehabilitation training systems and the transition from manual supervision to vision-based and generative paradigms are summarized in Figure 1.

### 1.3 The paradigm gap

This gap is intuitively reflected in the misalignment of task definitions. As noted in a systematic review by Lam et al. (12), existing vision algorithms predominantly focus on “Classification Tasks” (identifying *what* the user is doing), whereas the core requirement of rehabilitation lies in fine-grained “Action Quality Assessment (AQA)” (quantifying *how well* the user is doing) (13, 14). This necessitates algorithms with fine-grained spatiotemporal reasoning capabilities that transcend semantic categories. However, attempts to migrate general vision paradigms to this rigorous clinical scenario remain constrained by three core bottlenecks:

- **First, “Perception-Generalization Failure” in uncontrolled environments.** This bottleneck stems from the compound effects of “environmental interference,” “data bias,” and “mechanism defects.” On one hand, as pointed out by Lam et al. (12), existing models are mostly trained on “mimicked data” performed by healthy individuals, making it difficult to capture the pathological compensations of real patients (13). Consequently, systems are prone to failure when facing complex occlusions (e.g., wheelchairs) or unconventional postures (e.g., bedridden positions) in home settings (15). On the other hand, mainstream visual systems suffer from a deep “Ill-posed Nature” (16, 17)—deriving 3D pose from 2D images yields infinite solutions. Lacking “temporal context” to suppress high-frequency jitter and “biomechanical constraints” to ensure anatomical consistency, algorithms tend to generate predictions that are geometrically plausible but anatomically erroneous, termed **“Biomechanical Hallucinations”** (e.g., Bone Stretching, joint hyperextension). This fundamentally stems from simplifying the human body into a collection of isolated keypoints rather than a kinematic chain with rigid body properties, stripping the output of the robustness required for clinical measurement and highlighting the necessity of transitioning from keypoint inference to constructing a “Biomechanical Digital Twin” (18–20).
- **Second, the “Semantic Gap” in clinical assessment and reasoning logic.** This gap manifests hierarchically across three dimensions. (1) *Lack of Temporal Parsing:* Clinical assessment exhibits strict phase-dependency (e.g., knee control must be assessed separately during flexion and extension phases), yet traditional models often treat video as a flat sequence of frames. The lack of **“temporal semantic parsing”** capabilities (21) prevents the locking of correct biomechanical windows for calculation. (2) *Difficulty in Explicit Metric Translation:* Outputs of existing algorithms often remain as Implicit Visual Representations, which are difficult to decode into Explicit Clinical Metrics compliant with Evidence-Based Medicine (EBM) standards (e.g., ROM angles, movement smoothness SPARC/Jerk) (22). Consequently, clinicians cannot directly obtain quantifiable evidence with physical meaning. (3) *Absence of Pathological Attribution:* Systems lack a causal logic-based “pathological attribution mechanism” (23) and ignore the quantification of prediction uncertainty. They fail to identify specific **Compensatory Patterns** (e.g., shoulder hiking, compensatory trunk movement) like an expert, making it difficult to explain the root causes of poor movement quality and integrate into the clinical workflow.
- **Finally, the dual disconnect of “Guidance Precision” and “Rehabilitation Adherence” in feedback mechanisms (The Precision-Adherence Gap) (24).** On one hand (Precision), traditional feedback often remains at the level of mechanical “error reporting” or simple numerical scoring, lacking semantic translation based on Motor Learning Theory. Systems provide vague “Knowledge of Results (KR)” but fail to generate **“Knowledge of Performance (KP)”** to guide movement correction, leaving patients unable to rectify subtle compensatory errors. On the other hand (Adherence), existing interaction logic ignores patient pathological heterogeneity. Systems lack adaptive regulation of **“Cognitive Load,”** leading to information overload or inappropriate feedback timing. Furthermore, the absence of **“Multimodal Sensory Compensation”** mechanisms fails to make up for patients’ impaired proprioception. This cycle of inaccurate guidance and insufficient motivation may reduce training adherence, limit sensorimotor engagement, and weaken the motor-learning conditions required for effective rehabilitation.

### 1.4 Primary work

To bridge the aforementioned “Paradigm Gap” between General Vision and Clinical Application, this paper establishes a unified, end-to-end analytical framework grounded in the practical necessities of clinical rehabilitation: “**Perception, Assessment, and Coaching (PAC)**.” Using this framework as a logical grid, we systematically review and restructure the existing literature. Our objective is to elucidate the intrinsic connections between various technical components and provide a theoretical reference and technical roadmap for systematically addressing the challenges outlined above. This systematic literature review and the proposed PAC taxonomy are intended for a multidisciplinary audience, including computer vision researchers seeking to understand clinical constraints in medical applications, rehabilitation specialists and physiotherapists interested in how AI-driven tools can enhance assessment and coaching, and engineers developing intelligent home-based healthcare systems. Additionally, it serves as a technical reference for students and healthcare policy-makers concerned with the digital transformation of rehabilitation services. The framework is organized into three progressive Domains:

- **Perception Domain:** This Domain explores the technological evolution from geometric reconstruction to the **Biomechanical Digital Twin**. We analyze a robust pipeline composed of adaptive feature extraction, temporal lifting, and core constraint filtering. Specifically, we elucidate how introducing SMPL parametric human models (25, 26) and physical priors can mathematically eliminate “visual hallucinations” and suppress environmental interference, thereby ensuring the physical fidelity of the reconstruction results.
- **Assessment and Reasoning Domain:** Transcending traditional data fitting, this Domain establishes an interpretable clinical reasoning engine (27) aimed at bridging the “Semantic Gap.” We systematically describe the synergistic mechanism of three progressive sub-modules: temporal parsing, quality quantification, and pathological attribution. The focus is on locking biomechanical windows via temporal semantic parsing and utilizing explicit rules combined with implicit **manifold learning** to translate opaque features into explicit clinical metrics, thus achieving the leap from engineering parameters to **Evidence-Based Medicine (EBM)** evidence.
- **Coaching and Interaction Domain:** This Domain summarizes the technical architecture for constructing a closed sensorimotor intervention loop. We analyze how to achieve precise correction through the semantic translation of **“Knowledge of Performance (KP),”** addressing the ambiguity of traditional feedback. Furthermore, we explore the integration of the **“Feedback Priority Pyramid”** strategy (28) with immersive multimodal interaction technologies (29) (e.g., AR visualization and auditory mapping). The goal is to support motor relearning and long-term adherence through theory-informed multimodal feedback that is compatible with principles of motor learning and sensorimotor engagement.

### 1.5 Future outlook & contributions

Building upon this analytical model, this paper further envisions the frontier transformations driven by Artificial Intelligence. With the rapid advancement of Generative AI and Multimodal Large Language Models (MLLMs) (30), these technologies are beginning to permeate and reshape every component of the aforementioned taxonomy. In the latter half of this paper, based on the evolutionary trends in existing literature, we critically explore the application prospects of foundation models in the rehabilitation domain. We analyze how to leverage their powerful semantic reasoning capabilities to construct the system’s “**Cognitive Hub**,” thereby resolving the interpretability challenges of traditional models. Furthermore, we prospectively discuss the theoretical value and potential risks of Generative AI-driven “**Visual Self-Modeling (VSM)**” technology in future interaction paradigms.

The main contributions of this paper are summarized as follows:

- **Elucidated the task boundaries and paradigm differences between Rehabilitation Vision and General Vision:** We explicitly point out that the core challenge has shifted from “action classification” to “pathological movement quality quantification.” Furthermore, we demonstrate the necessity of introducing physiological priors and physical constraints to ensure the biomechanical consistency and anatomical realism of reconstructed results in uncontrolled environments.
- **Established a standardized three-Domain taxonomy based on a systematic literature review:** We synthesize the logical paradigm of **“Perception, Assessment, and Coaching (PAC),”** providing a structured perspective for analyzing the fragmented achievements of existing research.
- **Identified the evolutionary trajectory from “Discriminative Analysis” to “Generative Intervention”:** We deeply analyze the potential of Generative AI and MLLMs in reshaping semantic reasoning and interaction paradigms, offering forward-looking guidance for the development of next-generation intelligent **Rehabilitation Agents** equipped with autonomous logic.

## 2 Materials and methods

To ensure a comprehensive, transparent, and reproducible synthesis of the rapidly evolving landscape of vision-based rehabilitation, this review followed a systematic screening protocol grounded in the **PRISMA 2020** (Preferred Reporting Items for Systematic Reviews and Meta-Analyses) guidelines. The methodology was specifically designed to address the “Paradigm Gap” identified in the *Introduction* by intersecting engineering feasibility with clinical validity. In response to the methodological requirements of a systematic review, the research question, eligibility criteria, search strategy, evidence mapping, and methodological quality appraisal were further structured using a PICOS-informed framework and a PAC-based evidence synthesis strategy.

This review was not prospectively registered in PROSPERO. At the time of study design, the review was conceived as a multidisciplinary taxonomic and evidence-mapping review of computer vision-based rehabilitation systems, integrating engineering studies, clinical validation studies, rehabilitation prototypes, conference proceedings, reviews, and emerging AI-oriented works, rather than as a meta-analysis of a single clinical intervention or therapeutic effect. Nevertheless, the review was conducted according to a predefined search, screening, extraction, and evidence-mapping plan following the PRISMA 2020 reporting framework. The absence of prospective registration is acknowledged as a methodological limitation.

### 2.1 Research question and PICOS-informed framework

This review was guided by a PICOS-informed framework to clarify the systematic objectives, selection criteria, and scope of evidence synthesis. The overarching research question was: How have computer vision-based techniques been developed, validated, and translated across the Perception, Assessment, and Coaching stages of physical rehabilitation, and what methodological, clinical, and implementation gaps remain for their routine use in rehabilitation practice?

The PICOS elements were defined as follows:

- **Population (P):** Individuals undergoing or requiring physical rehabilitation, with particular attention to neurological rehabilitation populations (e.g., stroke, Parkinson’s disease), orthopedic rehabilitation populations (e.g., ACL reconstruction, total knee or hip arthroplasty, osteoarthritis), and geriatric or balance-related rehabilitation populations. Studies involving healthy participants were considered only when they were explicitly designed to simulate, validate, or benchmark rehabilitation-relevant movement assessment or coaching tasks.
- **Intervention/Technology (I):** Computer vision-based rehabilitation technologies, including markerless pose estimation, RGB or RGB-D motion analysis, 2D/3D human pose estimation, skeleton tracking, action quality assessment, compensatory movement detection, movement-quality quantification, visual feedback, biofeedback, and AI-assisted coaching systems.
- **Comparator (C):** Gold-standard or reference approaches, including optical motion capture systems such as VICON or OptiTrack, inertial measurement units (IMUs), Kinect or other depth sensors, goniometry, clinical scales, therapist ratings, expert annotations, or no comparator where the study was primarily conceptual, taxonomic, or technology-mapping in nature.
- **Outcomes (O):** Engineering and clinical outcomes, including Mean Per Joint Position Error (MPJPE), joint angle error, Range of Motion (ROM), Intraclass Correlation Coefficient (ICC), Root Mean Square Error (RMSE), movement smoothness, Jerk, Spectral Arc Length (SPARC), action quality scores, compensatory movement recognition, feedback precision, usability, adherence, safety, and clinical implementation indicators.
- **Study design (S):** Peer-reviewed empirical studies, technical validation studies, clinical validation studies, rehabilitation system prototypes, systematic or scoping reviews, high-quality conference proceedings, and selected frontier studies relevant to Generative AI and Multimodal Large Language Models (MLLMs). Frontier studies and preprints were used only to contextualize emerging directions and were not treated as equivalent to peer-reviewed clinical validation evidence.

### 2.2 Search strategy and information sources

A multidisciplinary search was conducted across eight major digital repositories to bridge computer science innovations with clinical rehabilitation medicine evidence. The search was categorized into three primary clusters:

- **Engineering & Computer Science:** **IEEE Xplore**, **ACM Digital Library**, and **arXiv** , with arXiv used specifically to capture emerging 2024–2026 Generative AI and MLLM frontier works rather than to support conclusions regarding established clinical effectiveness.
- **Medical & Life Sciences:** **PubMed/MEDLINE**, **Cochrane Library**, and **JMIR** (Journal of Medical Internet Research).
- **Comprehensive Multidisciplinary:** **Web of Science (Core Collection)** and **Scopus** were utilized to ensure coverage of high-impact journals from major publishers (Elsevier, Springer, Wiley, and MDPI).

Additionally, **Google Scholar** was employed for forward and backward citation tracing (snowballing) and to identify additional high-impact or frontier records not yet indexed in the primary databases.

The primary search horizon spanned from January 2010 to January 2026. The final search was conducted before manuscript preparation, and database-specific search strings, filters, search dates, and syntax adaptations are reported in Supplementary Data Sheet 1, Table S1. Search strings utilized Boolean operators to intersect the hierarchical domains of the PAC taxonomy:

1. **Domain A (Perception):** (“Human Pose Estimation” OR “3D Mesh Recovery” OR “Skeleton tracking”) AND (“Biomechanical Constraints” OR “Digital Twin” OR “SMPL” OR “Anatomical Prior”).
2. **Domain B (Assessment):** (“Action Quality Assessment” OR “AQA” OR “Movement evaluation”) AND (“Pathological Synergy” OR “Clinical Metrics” OR “Explainable AI” OR “EBM”).
3. **Domain C (Coaching):** (“Motor Learning” OR “Biofeedback” OR “Instructional feedback”) AND (“Multimodal Large Language Models” OR “MLLM” OR “Visual Self-Modeling” OR “Generative AI”).

To improve reproducibility, the search strategy was further operationalized using three concept blocks: rehabilitation context, computer vision technology, and assessment or coaching outcome. The general search structure was:

(“physical rehabilitation” OR “rehabilitation exercise” OR “stroke rehabilitation” OR “orthopedic rehabilitation” OR “home-based rehabilitation” OR “telerehabilitation”) AND (“computer vision” OR “human pose estimation” OR “markerless motion capture” OR “skeleton tracking” OR “3D pose estimation” OR “action quality assessment”) AND (“movement assessment” OR “clinical validation” OR “biofeedback” OR “coaching” OR “motor learning” OR “exercise feedback”).

This general structure was adapted to the controlled vocabulary and syntax requirements of each database. For example, PubMed/MEDLINE searches incorporated medical and rehabilitation terms, whereas IEEE Xplore and ACM Digital Library searches emphasized pose estimation, skeleton tracking, action quality assessment, and human-computer interaction terms. Web of Science and Scopus were used to capture multidisciplinary studies across engineering, rehabilitation medicine, digital health, and applied artificial intelligence.

All arXiv records and other non-peer-reviewed preprints were treated as frontier or emerging evidence. They were included only when they were directly relevant to rapidly evolving technical directions, such as Generative AI, MLLMs, foundation models, or frontier computer vision methods, and they were not weighted as equivalent to peer-reviewed clinical validation studies. Their evidence status was explicitly recorded during methodological appraisal and is reported in Supplementary Data Sheet 1, Table S2.

### 2.3 Inclusion and exclusion criteria

To ensure clinical fidelity and technical robustness, the following criteria were applied:

**Inclusion Criteria:** Studies were eligible if they met at least one core requirement within the PICOS-informed scope and contributed directly to the PAC framework. Specifically, eligible studies included: (1) peer-reviewed journal articles or top-tier conference proceedings (e.g., CVPR, ICCV, PeerJ CS, TNSRE); (2) research presenting vision-based systems specifically for neurological, orthopedic, or geriatric rehabilitation (2, 3, 31); (3) studies providing quantitative validation against “Gold Standard” ground truth (e.g., VICON, IMU) or EBM-compliant metrics (22, 32–34); (4) frontier works investigating foundation models for clinical reasoning (35–38); and (5) studies that contributed to at least one of the three PAC domains, namely Perception, Assessment, or Coaching.

**Exclusion Criteria:** (1) General Human Action Recognition (HAR) studies focusing solely on action classification without quality analysis (8, 9); (2) research relying exclusively on wearable sensors without a primary computer vision component; (3) **Metric-Utility Dissociation:** purely algorithmic studies optimizing engineering benchmarks (e.g., MPJPE) on static datasets without biomechanical consistency; (4) **Semantic Insufficiency:** works providing only summative “Knowledge of Results” (KR) without error attribution (KP) (39, 40); (5) studies unrelated to physical rehabilitation, motor function assessment, or rehabilitation-oriented movement coaching; and (6) studies for which the full text or essential methodological information could not be retrieved.

Because this review integrates both engineering and clinical literature, studies were not excluded solely because they lacked randomized clinical trial evidence. However, such studies were differentiated during evidence appraisal to avoid assigning equivalent evidential weight to preliminary prototypes, engineering benchmarks, and clinically validated systems.

### 2.4 Study selection and data extraction

After duplicate removal, titles and abstracts were screened according to the predefined PICOS-informed eligibility criteria. Full-text articles were then assessed for relevance to computer vision-based physical rehabilitation and mapped to the PAC framework. Reasons for exclusion at the title/abstract and full-text stages were documented and summarized in the PRISMA flow diagram.

During revision, two reviewers independently re-checked the title/abstract screening decisions, full-text eligibility decisions, and data extraction records against the predefined PICOS-informed criteria. Disagreements were resolved through discussion, and a senior reviewer was consulted when consensus was required. Because formal inter-rater reliability statistics were not prospectively recorded during the original screening process, Cohen’s kappa was not calculated for the initial screening and eligibility phases. This absence of prospectively recorded inter-rater reliability statistics is acknowledged as a methodological limitation of the review.

For each included study, key information was extracted using a standardized data extraction form. Extracted items included: author and year, publication type, rehabilitation population, study design, vision modality, algorithmic method, PAC domain, comparator or reference standard, validation metric, clinical outcome or movement-quality indicator, feedback or coaching strategy, real-world deployment context, evidence status, and main limitations. This structured extraction process supported both descriptive synthesis and PAC-based evidence mapping.

### 2.5 Methodological quality appraisal and evidence grading

To address methodological heterogeneity and avoid discussing all publications as if they provided the same level of evidence, a customized methodological quality appraisal framework was developed for this review. Conventional tools such as QUADAS-2 or ROBINS-I are designed for specific diagnostic or observational study designs and are not fully suitable for a mixed corpus that includes clinical validation studies, engineering benchmarks, rehabilitation prototypes, conference proceedings, and emerging AI-oriented works. Therefore, a customized checklist was used to assess methodological robustness and evidence strength across the included studies.

The quality appraisal considered the following dimensions:

1. **Population specificity:** whether the study involved actual rehabilitation patients or only healthy participants performing simulated tasks.
2. **Vision-based relevance:** whether computer vision, RGB/RGB-D video, markerless pose estimation, or skeleton tracking was a primary component of the system.
3. **Reference standard:** whether the study used VICON, OptiTrack, IMU, Kinect, goniometry, clinical scales, therapist ratings, or other reference standards.
4. **Clinical metric reporting:** whether clinically meaningful metrics such as ROM, ICC, RMSE, Jerk, SPARC, movement smoothness, compensatory movement detection, or action quality score were reported.
5. **External or independent validation:** whether the model or system was evaluated using an independent dataset, external cohort, or cross-setting validation.
6. **Real-world testing:** whether the study was conducted in home-based, remote, community, or otherwise uncontrolled environments rather than only in laboratory settings.
7. **Interpretability and error attribution:** whether the system provided clinically interpretable outputs, compensatory-pattern localization, or explanatory feedback rather than only black-box scores.
8. **Evidence status:** whether the work was a peer-reviewed journal article, peer-reviewed conference proceeding, systematic review, prototype study, protocol, benchmark-only study, or non-peer-reviewed frontier preprint.

Accordingly, this customized framework was used as an evidence-mapping and evidence-weighting instrument rather than as a conventional risk-of-bias tool for a single study design. Its purpose was to distinguish clinically validated systems from engineering benchmarks, prototypes, reviews, protocols, and emerging preprints, rather than to generate a pooled risk-of-bias score across methodologically incompatible study designs.

Each record was classified according to PAC domain, use in review, rehabilitation population or context, study role, reference standard or validation metric, validation context, evidence status, and overall appraisal category. The overall appraisal categories included High, Moderate to high, Moderate/technical, Secondary evidence, Emerging, and Contextual. The complete methodological quality appraisal and evidence weighting are provided in Supplementary Data Sheet 1, Table S2. Studies were not excluded solely on the basis of quality appraisal because the purpose of this review was to map the technological and clinical landscape of vision-based rehabilitation. However, the appraisal results were used to contextualize the strength of evidence and to distinguish clinically validated systems from preliminary or conceptual works.

To avoid conflating heterogeneous evidence types, explicit evidence hierarchies and interpretation boundaries were applied during synthesis. Studies with direct patient-level validation, randomized or controlled designs, biomechanical or clinical reference standards, clinically relevant comparators, or therapist-rated outcomes were interpreted as stronger evidence for clinical readiness. Engineering benchmark studies were used primarily to discuss algorithmic feasibility, technical performance, and methodological limitations. Reviews and contextual/background papers were used to frame the field and support background interpretation, whereas protocols, preprints, benchmark-only studies, and frontier AI studies were treated as emerging or contextual evidence. These records were retained when relevant to the PAC taxonomy or future technical trajectories, but they were conservatively weighted and were not used as primary support for established clinical effectiveness or routine clinical deployment.

In particular, non-peer-reviewed preprints and rapidly evolving frontier AI studies were explicitly labeled as Emerging evidence in the appraisal process. They were used to contextualize future technical trajectories, especially in relation to MLLMs, Generative AI, and foundation-model-assisted rehabilitation, but were not used as primary evidence for clinical effectiveness or routine clinical readiness.

### 2.6 Selection results and data mapping

**Figure 2:**
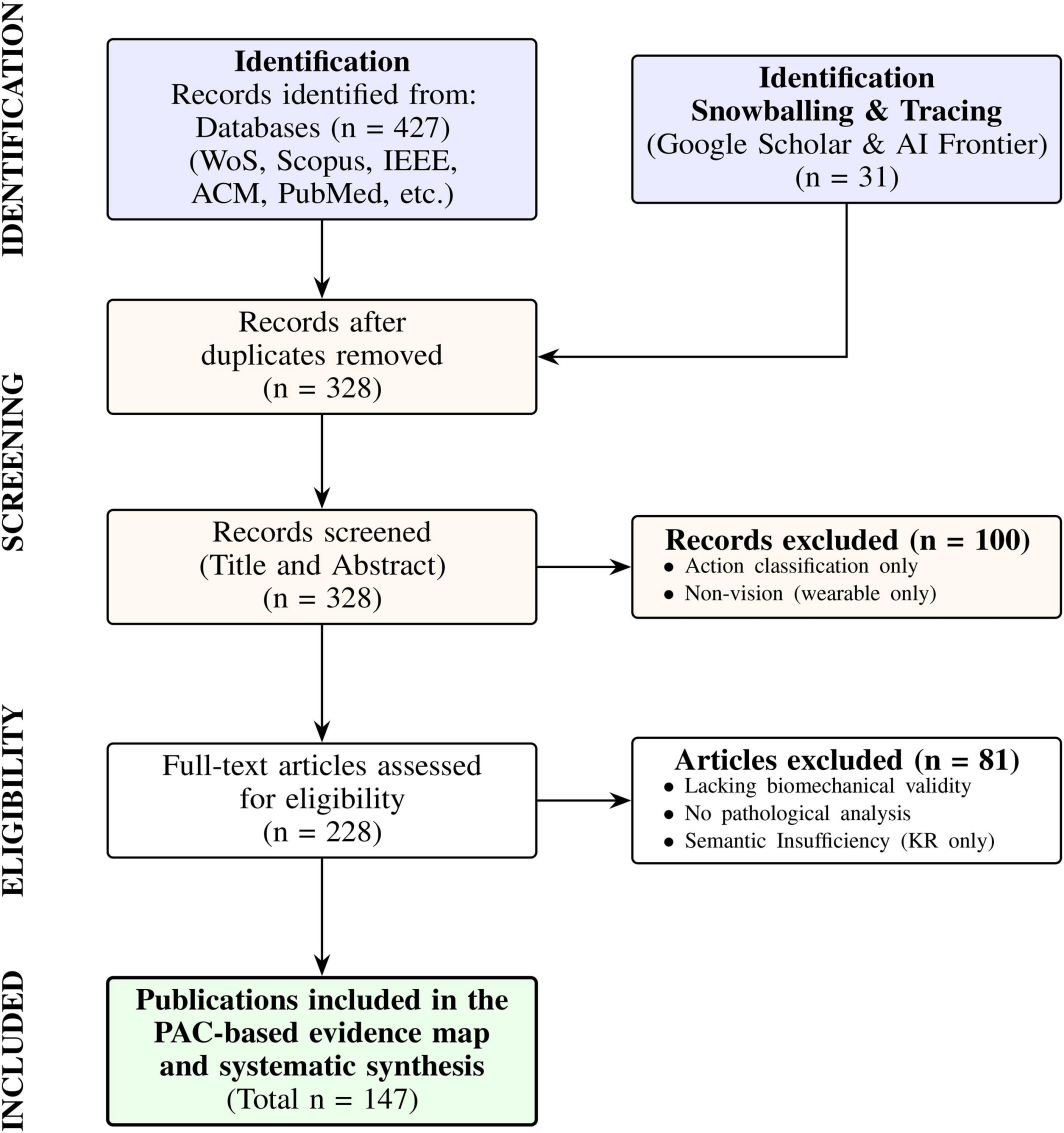
PRISMA 2020 flow diagram. The strategy incorporates WoS, Scopus, Google Scholar, and frontier tracing to ensure a comprehensive multidisciplinary synthesis, yielding 147 publications for PAC-based evidence mapping and methodological appraisal.

The systematic workflow is illustrated in Figure 2. The primary database search identified 427 records, while frontier tracing and Google Scholar snowballing added 31 high-impact records, totaling 458. After removing 130 duplicates and screening 328 titles/abstracts, 228 articles were assessed for full-text eligibility. Finally, **147 publications** were included in the PAC-based evidence map and systematic synthesis. The included studies were subsequently mapped according to PAC domain, rehabilitation population, study design, validation method, evidence status, and evidence level. This mapping enabled the review to synthesize not only the technical evolution of computer vision-based rehabilitation but also the methodological robustness, peer-review status, and clinical relevance of the available evidence.

Compared with a purely narrative synthesis, this PAC-based evidence mapping allowed the included publications to be interpreted according to their functional role: Perception studies primarily addressed movement capture and biomechanical reconstruction; Assessment studies focused on movement-quality quantification, clinical metrics, and pathological attribution; and Coaching studies examined feedback delivery, motor learning, and patient-facing interaction strategies. The methodological quality appraisal was used alongside this mapping to avoid assigning equal evidential authority to engineering prototypes, preliminary feasibility studies, non-peer-reviewed preprints, and clinically validated systems.

To further reduce reliance on narrative synthesis alone, we summarized the included records using a descriptive quantitative evidence map. This summary reports the distribution of records across PAC domains, technology-related functional categories, rehabilitation populations or pathological contexts, evidence status, and overall appraisal levels. Because the included publications were highly heterogeneous in terms of study design, technology modality, comparator, population, and outcome metrics, formal meta-analysis was not appropriate. Instead, the descriptive evidence map was used to identify where the available evidence is concentrated and where important gaps remain (Table 1). The detailed record-level coding is provided in Supplementary Data Sheet 1, Table S2.

**Table 1:** Descriptive quantitative evidence map of the 147 included records.

Mapping dimension	Distribution of included records	Main implication
PAC domain	Perception only: 56 (38.1%); Assessment only: 20 (13.6%); Coaching only: 16 (10.9%); Cross-domain PAC records: 19 (12.9%); PAC-related technical/contextual evidence: 29 (19.7%); Context/background evidence: 7 (4.8%).	Evidence is concentrated in Perception.
Technology-related functional focus	Perception-related visual measurement, pose estimation, skeleton tracking, or reconstruction: 71 (48.3%); Assessment-related AQA, movement-quality scoring, clinical metric extraction, or compensatory-pattern detection: 36 (24.5%); Coaching-related feedback, biofeedback, multimodal interaction, or AI-assisted guidance: 25 (17.0%).	Closed-loop coaching remains less represented.
Population or pathology	General, benchmark, healthcare-context, or not population-specific: 88 (59.9%); General rehabilitation exercise, physiotherapy, upper-limb function, or home-based contexts: 22 (15.0%); Neurological rehabilitation: 21 (14.3%); Orthopedic or musculoskeletal rehabilitation: 9 (6.1%); Geriatric, gait, balance, or mobility contexts: 6 (4.1%); Pediatric rehabilitation: 1 (0.7%).	Disease-specific rehabilitation evidence is limited.
Evidence status	Peer-reviewed journal articles: 82 (55.8%); Peer-reviewed conference proceedings: 36 (24.5%); Secondary evidence: 17 (11.6%); Preprint or emerging evidence: 10 (6.8%); Other publication types: 2 (1.4%).	Most records are peer-reviewed, but emerging evidence remains present.
Overall appraisal level	High: 0 (0.0%); Moderate to high: 10 (6.8%); Moderate or moderate/technical: 68 (46.3%); Technical benchmark or contextual technical evidence: 24 (16.3%); Secondary evidence: 17 (11.6%); Emerging evidence: 13 (8.8%); Contextual evidence: 15 (10.2%).	High-level clinical validation evidence remains scarce.

Counts are based on conservative record-level coding in Supplementary Data Sheet 1, Table S2. Percentages use 147 included records as the denominator. PAC-domain, population/pathology, evidence-status, and overall-appraisal categories are presented as simplified descriptive summary groups. Technology-related functional focus categories are not mutually exclusive because cross-domain records may contribute to more than one PAC function. This table is intended as descriptive quantitative synthesis rather than meta-analysis.

Overall, the descriptive evidence map indicates that the current literature remains concentrated in Perception-oriented visual measurement and reconstruction, whereas Assessment and especially Coaching studies are less represented. It also shows that many records remain general, benchmark-oriented, or not population-specific, and that high-level clinical validation evidence remains limited.

## 3 Clinical context and technical challenges

While computer vision technologies have reached a high level of maturity in general Human Action Recognition (HAR), migrating them to medical rehabilitation scenarios continues to confront a significant **“Clinical Adaptability Gap.”** The essence of rehabilitation transcends mere mechanical repetition; rather, it is a process of remodeling neuromuscular pathways under pathological constraints. To construct visual systems endowed with *Clinical Validity*, we must transcend pure algorithmic optimization and deeply deconstruct the authentic clinical context in which these algorithms operate. This section dissects the unique challenges facing rehabilitation vision from three core dimensions: pathological characteristics, quantification requirements, and environmental constraints. The mapping between clinical requirements, computer vision challenges, and validity criteria is summarized in Table 2.

**Table 2:** Mapping clinical requirements to computer vision challenges and validity criteria.

Clinical domain & Population	Key biomarkers (clinical focus)	CV translation (technical tasks)	Specific bottlenecks (domain gap)	Validity requirements (success criteria)
Orthopedic Rehab (TKA, ACL, OA)	Geometric Alignment; Valgus/Asymmetry; ROM Recovery (41, 42)	High-fidelity 3D Reconstruction; Bone Length Consistency; Viewpoint Invariance	**Perspective Foreshortening & Scale Ambiguity:** 2D estimation fails in non-canonical views (e.g., supine). *Sol: Parametric models (33)*	**Geometric Acc.:** Error $<5^{\circ }-10^{\circ }$; ICC >0.85 (31, 43)
Neurological Rehab (Stroke, Parkinson’s)	Motor Control Quality; Smoothness; Pathological Synergy (44)	Fine-grained Temporal Modeling; Trajectory Analysis; Functional Task	**Jitter Noise Amplification:** Micro-tremors indistinguishable from sensor noise (45); Risk of cascade failure (46)	**Temporal Stability:** Capture trajectory smoothness; Distinguish tremors (>5Hz) (47)
Geriatric & Balance (Fall, Sarcopenia)	Postural Stability; CoM vs. BoS Relationship; TUG (48, 49)	Physics-aware Pose Estimation; CoM Tracking; Dynamic Posture	**Environment & Occlusion:** Dynamic occlusion (walkers) requires long-range modeling (50); Lack of physics constraints (51)	**Occlusion Robustness:** Accurate CoM prediction under self-occlusion; Explainable determinants (52)
Safety & Compensation (Cross-population)	Compensatory Patterns; Shoulder Hiking; Trunk Lean (53)	Multi-label Anomaly Detection; Attribute Recognition; Reasoning	**Subtle “Cheating”:** Lack of OOD pose data (54, 55); Data augmentation for rare errors (56)	**Interpretability:** Pinpoint compensatory joints via XAI (57); Real-time feedback latency.

This table maps high-level clinical requirements to specific CV tasks detailed in the *Clinical Context and Technical Challenges* section.

TKA, total knee arthroplasty; ACL, anterior cruciate ligament; ROM, range of motion; ICC, intraclass correlation coefficient; CoM, center of mass; BoS, base of support; TUG, timed up and go; XAI, explainable AI; OOD, out-of-distribution.

### 3.1 Typical rehabilitation populations and kinematic characteristics

Unlike the pursuit of maximal strength or explosive power common in general fitness populations, the kinematic characteristics of rehabilitation patients are primarily defined by restricted joint Range of Motion (ROM), diminished motor control capabilities, and pathological synergy patterns. Existing vision-based rehabilitation research encompasses a full age spectrum ranging from neurodegenerative pathologies to musculoskeletal injuries.

#### 3.1.1 Neurological rehabilitation: focusing on coordination and pathological synergy

Patients with stroke or Parkinson’s disease (58) face primary challenges stemming from motor control disorders caused by Central Nervous System (CNS) damage.

- **Clinical Features & Technical Challenges:** When performing Activities of Daily Living (ADL), such as “Reach-to-grasp” tasks (59), hemiplegic patients often exhibit abnormal **Co-contraction** or **Pathological Synergy Patterns** (e.g., the upper limb flexor synergy, where an attempt to elevate the shoulder triggers involuntary elbow flexion). While research in this domain is active—for instance, SmartRehab (43) captures trajectory smoothness, and the SARAH system (60) attempts to quantify control deficits in functional tasks—mainstream action recognition models (e.g., I3D (61), SlowFast (62)) remain limited. These models typically rely on the “global pooling” of spatiotemporal features, excelling at classifying broad action categories but lacking explicit modeling of local topological relationships between joints. Consequently, algorithms struggle to distinguish “healthy compensation” from “pathological synergy” (e.g., differentiating “active elbow flexion” from “passive flexion induced by Associated Reactions”). Capturing these subtle motor control features far exceeds the scope of simple classification tasks, indicating that future intelligent rehabilitation systems must possess **“Temporal Semantic Parsing”** capabilities to finely isolate features of impaired neural control from continuous motion streams.

#### 3.1.2 Orthopedic rehabilitation: focusing on geometric alignment and weight bearing

For patients undergoing Total Knee/Hip Arthroplasty (TKA/THA) or Anterior Cruciate Ligament (ACL) reconstruction, the core of rehabilitation lies in restoring ROM and correcting biomechanical alignment.

- **Clinical Features & Technical Challenges:** During squats or Sit-to-Stand transfers, patients often exhibit bilateral **Asymmetry** or Knee **Valgus** due to pain or muscle weakness. Recent works like Motion Coach (63) and HGcnMLP (64) have attempted to monitor movement quality in Osteoarthritis (OA) patients, while Kryeem et al. (65) developed a multi-label feedback system for post-hip arthroplasty. However, it is imperative to scrutinize that existing general vision algorithms (e.g., HRNet (66), ViTPose (67)) fundamentally optimize “pixel-level probability heatmaps” rather than “geometric structural consistency.” In the absence of rigid skeletal constraints, the limb lengths output by these models often exhibit unnatural temporal **“Bone Stretching,”** which significantly amplifies joint angle errors calculated based on vectors. This rigorous requirement for **Geometric Alignment** renders the depth ambiguity common in general models unacceptable. This directly leads to a key design criterion: systems must introduce strict **“Biomechanical Constraints”** to correct visual geometric distortions and ensure the anatomical validity of ROM measurements.

#### 3.1.3 Geriatric & balance rehabilitation: focusing on postural stability

For the elderly population at high risk of falling, clinical assessments often rely on the Timed Up and Go (TUG) test or single-leg stance to evaluate postural control.

- **Clinical Features & Technical Challenges:** Visual systems must prioritize the dynamic relationship between the Center of Mass (CoM) and the Base of Support (BoS). He et al. (68) validated the efficacy of TUG tests for sarcopenia patients, and the KIMORE dataset (32, 69) demonstrated balance exercise monitoring. However, a fundamental dilemma must be addressed: precise stability analysis depends on the accurate 3D spatial relationship between the CoM and BoS. Existing 3D Lifting algorithms universally suffer from “depth uncertainty,” frequently resulting in reconstructed skeletons that exhibit **“Foot Sliding”** or “Floating” phenomena. On such reconstruction results lacking stable **Ground Contact**, any calculation regarding dynamic balance loses its physical meaning. This demand for perceiving physical contact and gravity vectors guides the next generation of visual algorithms to introduce **“Physics-aware Constraints”** (e.g., gravity direction, Ground Reaction Forces) aimed at transforming visual output from “floating geometric skeletons” into “grounded physical entities,” thereby guaranteeing the clinical fidelity of balance assessment.

### 3.2 From clinical scales to visual metrics: multidimensional quantification of movement quality and safety

The core of “Precision Coaching” lies in translating ambiguous clinical observations into computable mathematical metrics. Although existing clinical assessment systems [e.g., Fugl-Meyer Assessment (FMA), Berg Balance Scale (BBS)] have established a set of evaluation standards covering basic parameters such as gait speed, stride length, and symmetry, and computer vision has achieved broad coverage of these metrics in general motion analysis, mere basic kinematic parameters are often insufficient to reveal the pathological essence within the specific clinical context of rehabilitation. To construct a visual system with clinical diagnostic validity, this section focuses on three core and technically challenging dimensions—“Geometric Structural Integrity,” “Temporal Control Quality,” and “Pathological Compensation Mechanisms”—to deeply analyze the mapping gap between existing visual metrics and clinical gold standards.

#### 3.2.1 Geometric metrics: geometric alignment and consistency

Range of Motion (ROM) serves as the “Gold Standard” for evaluating rehabilitation progress.

- **Challenges & Technical Limitations:** ROM measurement under mainstream visual systems is limited by **Perspective Foreshortening**. Although SmartRehab (43) and HGcnMLP (64) have reported high Intraclass Correlation Coefficients (ICC >0.8) with clinical gold standards (e.g., VICON/Goniometer), benchmark tests on the UCO dataset (70) indicate that angular measurement errors of general pose estimation models can still exceed clinically acceptable ranges ($>5^{\circ }-10^{\circ }$) under specific viewpoints (e.g., non-frontal) or postures (e.g., supine). Fundamentally, existing mainstream algorithms [e.g., HRNet (66), ViTPose (67)] optimize “pixel-level probability heatmaps” rather than “geometric structural consistency.” In the absence of rigid skeletal constraints, the limb lengths output by these models often exhibit unnatural temporal **“Bone Stretching,”** which significantly amplifies joint angle errors calculated based on vectors. This indicates that simple pixel-level fitting cannot meet the Clinical Gold Standard. To fundamentally resolve the foreshortening issue, visual perception must evolve towards a **“Biomechanical Digital Twin,”** utilizing prior knowledge to reconstruct authentic anatomical angles in 3D space.

#### 3.2.2 Temporal quality metrics: smoothness and jitter suppression

Movement Smoothness is a critical biomarker for neural recovery.

- **Algorithm Implementation & Limitations:** Clinically common metrics such as “Jerk” or “Spectral Arc Length (SPARC)” are essentially calculations of the second or third derivatives of position data. SmartRehab (43) explicitly integrated the Jerk metric to monitor Spasticity. However, it must be noted that current lightweight on-device models (e.g., MediaPipe (71), BlazePose (72)) are inherently based on single-frame prediction, and their native outputs typically contain 3–5 mm of high-frequency Gaussian noise (**Jitter**) before filtering. This minute positional error undergoes **“Catastrophic Amplification”** during the differentiation process, resulting in generated Jerk curves full of artifacts that completely mask the patient’s true movement tremors. This rigorous requirement for high-frequency signal stability directly establishes a technical imperative: the system must introduce **“Temporal 3D Reconstruction”** and smoothing mechanisms to effectively suppress temporal noise that interferes with clinical measurement, ensuring the computational fidelity of high-order kinematic metrics.

#### 3.2.3 Compensation and safety: multi-label detection and attribution

Compensatory movements are mechanisms where patients utilize incorrect muscle groups to complete tasks (e.g., shoulder hiking instead of arm lifting).

- **Multi-label Detection & Limitations:** Unlike standard action recognition, compensation is often a “micro-error posture” superimposed on a “correct action.” Pornpipatsakul et al. (53) developed a detection algorithm specifically for “excessive knee width” in stroke Bridging exercises; Marusic et al. (73) proposed a Transformer-based multi-label error localization model for lower back pain. Regrettably, existing mainstream AQA models (typically based on MSE loss for end-to-end regression) tend to learn the “average features” of an action to optimize the overall score. This means that **“Outlier”** compensatory postures located at the edges of the feature distribution are often smoothed out by the model as noise, leading to a high false-negative rate for early, subtle compensations. This demand for attributing “micro-errors” profoundly reveals the **semantic gap** existing in current **opaque end-to-end models**. Therefore, an effective assessment module requires not just scoring, but the establishment of an interpretable **“Pathological Attribution Diagnosis”** mechanism, thereby explicitly decoding implicit features into specific compensatory patterns of concern to clinicians.

### 3.3 Real-world constraints and domain gaps in home and remote scenarios

Transferring Computer Vision (CV) algorithms from controlled laboratory settings to authentic home-based rehabilitation scenarios (“In-the-wild”) confronts significant **“Sim-to-Real”** domain shifts. This has become a focal point of recent literature and represents the primary hurdle that the “Perception Domain” must resolve.

#### 3.3.1 Environmental noise: uncontrolled backgrounds and occlusions

Home environments are typically characterized by cluttered backgrounds, variable lighting, and confined spaces. Research on the SARAH system (60) highlights the immense challenges posed by low frame rates, motion blur, and non-expert camera angles. Confined spaces often lead to **Truncation**, while a single fixed camera angle (usually frontal or oblique) causes severe **Self-occlusion**.

- **Domain Drift & Technical Limitations:** It must be noted that mainstream Pre-trained Models [e.g., OpenPose (74), AlphaPose (75)] are predominantly trained on datasets with relatively clean or controlled backgrounds, such as COCO or Human3.6M (34). When migrated to home environments filled with household objects (e.g., sofas, stacked clothes), models are prone to **“Texture Confusion,”** mistaking background textures for limbs. This triggers **“Biomechanical Hallucinations”** that violate physical laws (e.g., limb interpenetration, reverse bending).
- **Solution Strategy:** To address this pain point, clinical-grade algorithms require a specialized **“Biomechanical Constraint Filtering”** mechanism. This mechanism serves as a **“Biomechanical Firewall”** for purely data-driven algorithms, enforcing physical priors—such as anti-penetration and joint angle limits—to rigorously correct errors that violate anatomical common sense.

#### 3.3.2 Interaction & viewpoint challenges: patient-centric design

This is one of the primary causes for the failure of general pre-trained models, directly impacting the interaction robustness of the system.

- **Body Position Heterogeneity:** Rehabilitation training often involves lying, sitting, or quadruped positions. A systematic evaluation by Aguilar-Ortega et al. (70) on the UCO dataset demonstrated that State-of-the-Art (SOTA) pose estimation models suffer a precipitous performance drop when processing **Supine Poses**.
- **Critique of Training Distribution Bias:** The root of this failure lies in the severe **“Long-tailed Distribution”** problem of general datasets [e.g., Human3.6M (34)]. The vast majority of samples in these datasets are upright walking actions, causing models to learn a strong **“Upright Prior.”** When tailored to rehabilitation-specific postures like lying or crawling, models often erroneously interpret them as “standing actions viewed from a top-down angle,” resulting in severe pose distortions.

#### 3.3.3 Device & operational load: reducing cognitive barriers

Although studies by Pereira (76) and Cunha (77) have proven the feasibility of ubiquitous smartphone-based solutions, they also emphasize the system’s **Viewpoint Dependence**. If algorithms lack **View-invariant** 3D reconstruction capabilities (41, 76), patients are forced to strictly adhere to specific shooting angles.

- **Limitations of Projection Assumptions:** It is crucial to point out that existing 3D reconstruction algorithms (e.g., HMR, SPIN) typically rely on the **“Weak Perspective Projection”** assumption (78), which presumes the camera is positioned directly in front of or slightly to the side of the subject. Once the home shooting angle changes drastically (e.g., placed on the floor shooting up, or high up shooting down), this assumption fails, leading to severe flattening of the reconstructed human pose in the depth dimension. This not only increases the patient’s **Operational Load** and cognitive burden but also weakens adherence to long-term use. This indicates that future interaction and coaching modules must possess **“Adaptive”** capabilities to lower the cognitive and operational thresholds for patients, achieving a truly “Patient-Centric” design.

To summarize, the aforementioned **Cascading Challenges**—stemming from pathological heterogeneity, quantification fidelity, and uncontrolled environments—not only reveal deep-seated defects in the clinical adaptability of existing vision technologies but also establish rigid “Clinical Design Criteria.” In light of these challenges, this review introduces the unified **“Perception, Assessment, and Coaching” (PAC) taxonomy** as an analytical model. This taxonomy aims to systematically categorize the literature’s responses to these real-world barriers and map the technical evolution from algorithmic prototypes to clinically validated applications.

## 4 A taxonomy and logical architecture for vision-based rehabilitation

**Figure 3:**
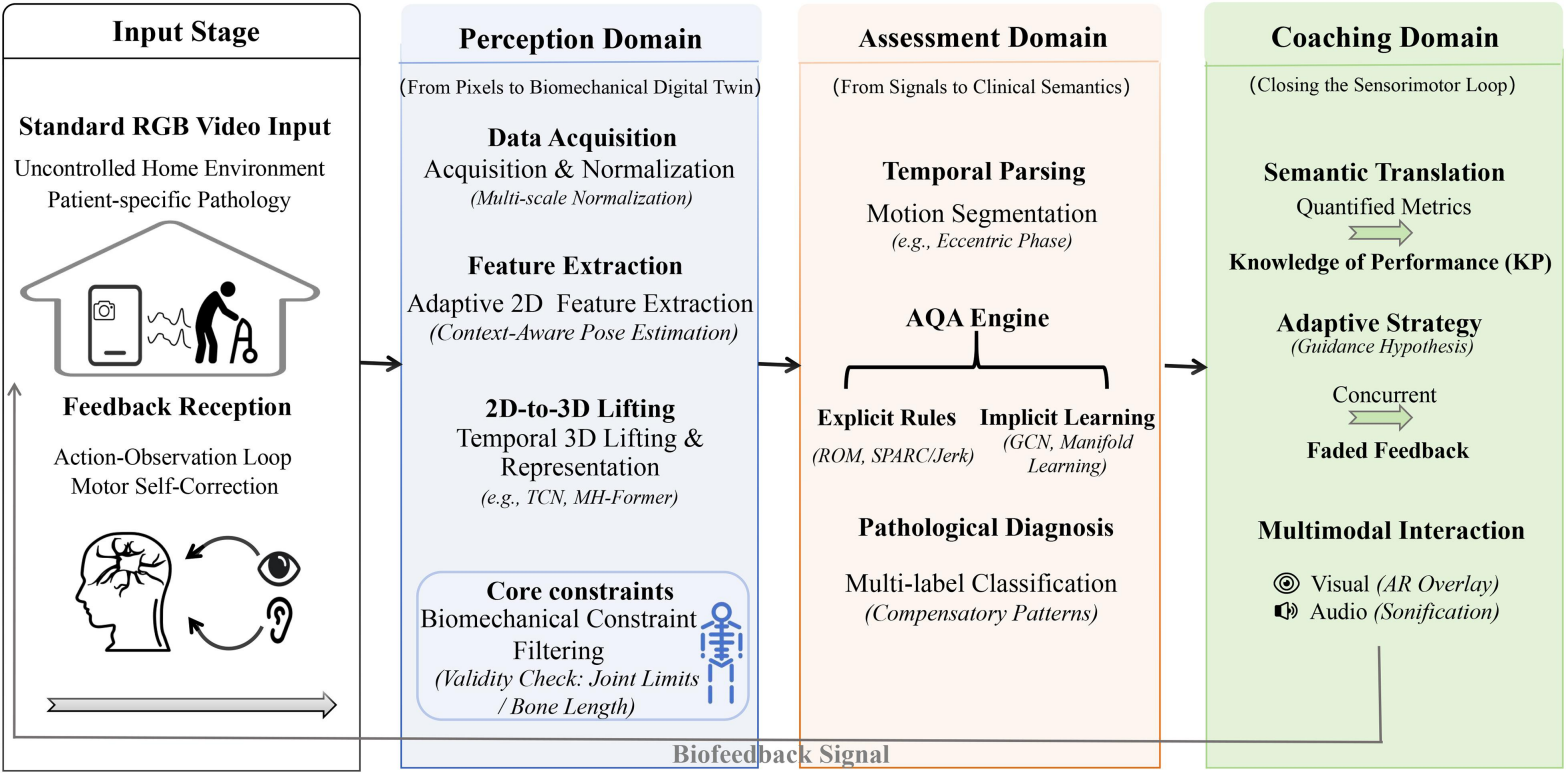
Taxonomy and methodological map of the vision-based rehabilitation landscape: the PAC analytical model. This schematic synthesizes the technological trajectories identified across the 147 reviewed publications, categorizing diverse methodologies into three synergistic research domains: **(1) Perception Domain:** Current research directions in this category prioritize the construction of high-fidelity “Biomechanical Digital Twins” by integrating anatomical constraints to ensure physical validity, a trend observed as a primary solution to visual hallucinations in recent literature. **(2) Assessment Domain:** Methodologies reviewed in this tier aim to resolve the semantic gap by quantifying movement quality through temporal semantic parsing and hybrid AQA (Action Quality Assessment) strategies documented in current studies. **(3) Coaching Domain:** Paradigms in this category explore strategies for closing the sensorimotor loop, focusing on the translation of quantitative metrics into actionable Knowledge of Performance (KP) to support motor relearning and patient-facing behavioral feedback. The dashed line illustrates the biofeedback loop identified in recent clinical-technical paradigms, serving as a **behavioral trigger mechanism** that influences motor intent and kinematic distribution, as synthesized from the reviewed literature.

To bridge the **“Semantic Gap”** identified in the *Clinical Context and Technical Challenges* between raw pixel-level data and high-level clinical decision-making, this review formulates a hierarchical analytical model and taxonomy—**“Perception, Assessment, and Coaching (PAC)”** (see Figure 3). Serving as a structural grid, this taxonomy delineates the systematic, end-to-end technical pipeline observed in recent literature, which aims to transform uncontrolled visual perception into interpretable reasoning and, ultimately, closed-loop clinical intervention. This section elucidates the underlying logic of this taxonomy, establishing a theoretical blueprint to systematically categorize and review the fragmented existing literature in subsequent sections. **Positioning of the PAC taxonomy relative to existing frameworks.** To clarify the originality and scope of the PAC taxonomy, Table 3 compares PAC with existing clinical, methodological, computer-vision, action-quality-assessment, motor-learning, and implementation-oriented frameworks. PAC is not intended to replace PRISMA, PICOS, ICF, conventional computer-vision pipelines, action-quality-assessment models, motor-learning theory, or implementation-oriented regulatory frameworks. Rather, it integrates these complementary perspectives into a domain-specific analytical structure for vision-based rehabilitation systems by linking visual measurement, clinical interpretation, and patient-facing feedback.

**Table 3:** Positioning of the PAC taxonomy in relation to existing conceptual and methodological frameworks.

Framework	Primary focus	Limitation for vision-based rehabilitation	Added value of the PAC taxonomy
PRISMA/PICOS	Provides reporting standards and eligibility logic for systematic reviews, including population, intervention, comparator, outcomes, and study design.	Useful for transparent review conduct, but it does not provide a domain-specific structure for explaining how video-derived data are transformed into clinical assessment and therapeutic feedback.	PAC complements PRISMA/PICOS by serving as the analytical taxonomy for evidence synthesis, organizing heterogeneous studies according to their functional role in rehabilitation systems: perception, assessment, and coaching.
ICF-oriented rehabilitation frameworks	Describe functioning, disability, activity, participation, and environmental factors from a clinical and biopsychosocial perspective.	Clinically comprehensive, but not designed to classify computer-vision pipelines, pose-estimation validity, algorithmic assessment, or closed-loop feedback mechanisms.	PAC translates rehabilitation needs into computational stages, linking movement capture to clinical interpretation and patient-facing intervention while remaining compatible with broader functional-outcome frameworks.
Conventional computer-vision pipeline	Focuses on image/video acquisition, detection, pose estimation, tracking, action recognition, and benchmark accuracy.	Primarily engineering-oriented; it often emphasizes metrics such as mAP, PCK, or MPJPE without directly addressing clinical validity, pathological compensation, feedback safety, or rehabilitation adherence.	PAC extends the computer-vision pipeline toward clinical translation by requiring biomechanically valid perception, clinically interpretable assessment, and actionable coaching rather than pose accuracy alone.
Action Quality Assessment frameworks	Evaluate how well an action is performed by estimating movement quality scores, error patterns, or performance levels.	AQA frameworks bridge action recognition and movement-quality scoring, but they often remain centered on assessment and do not fully specify how assessment outputs should be converted into safe, individualized rehabilitation feedback.	PAC incorporates AQA within the Assessment domain and further connects it to Perception validity and Coaching delivery, thereby supporting a full measurement–reasoning–feedback loop.
Motor-learning and feedback theories	Explain feedback type, timing, frequency, knowledge of results, knowledge of performance, cognitive load, retention, and skill acquisition.	Highly relevant to rehabilitation coaching, but they do not specify how visual sensing, pose reconstruction, or algorithmic movement-quality assessment should be validated before feedback is delivered.	PAC embeds motor-learning principles within the Coaching domain while preserving the upstream requirements of accurate perception and clinically meaningful assessment.
Digital health/SaMD implementation frameworks	Address intended use, clinical validation, risk management, usability, cybersecurity, regulatory readiness, and post-market monitoring.	Important for translation and governance, but usually not organized around the technical sequence from video input to movement interpretation and corrective feedback.	PAC provides a system-level map that helps identify which component of a vision-based rehabilitation system requires validation, risk control, and regulatory evidence before clinical deployment.

### 4.1 The perception domain: from geometric reconstruction to biomechanical digital twins

Acting as the “eyes” of intelligent rehabilitation systems, research within the Perception domain is increasingly transcending the mere pursuit of standard MPJPE benchmarks, shifting focus toward constructing high-fidelity digital twins with **Biomechanical Consistency** (19, 33). To address the significant “Sim-to-Real” domain shift, current state-of-the-art literature can be categorized into a four-stage pipeline:

- **Acquisition & Normalization:** Methods in this phase address the randomness of home recording viewpoints by performing geometric rectification, a step crucial for mitigating the **Scale Ambiguity** and perspective distortions inherent in single-view projections (70, 79).
- **Adaptive Feature Extraction:** Designed to resolve environmental interference, studies in this category extract noise-resistant anatomical keypoints (8) rather than simple coordinates, ensuring robustness against the complex occlusions and non-canonical poses typical in home-based rehabilitation (72).
- **Temporal 3D Lifting:** Targeting the instability of single-frame predictions, temporal lifting techniques utilize temporal context (80) and inter-frame consistency constraints to suppress **Jitter** and resolve the ill-posed nature of mapping 2D observations to 3D mesh representations (81).
- **Biomechanical Constraint Filtering:** As a critical frontier, recent approaches integrate SMPL parametric models (25) with human dynamics priors (e.g., constant bone length) to mathematically eliminate **“Visual Hallucinations”** and ensure the reconstructed output is anatomically authentic (82).

### 4.2 The assessment domain: from opaque mapping to clinical attribution reasoning

Functioning as the “brain” of the rehabilitation pipeline, research in the Assessment domain shifts the focus from simple action classification (12) to fine-grained **Action Quality Assessment (AQA)** and pathological reasoning (14). Within this review, this reasoning process is abstracted into three progressive categories:

- **Phase 1: Temporal Semantic Parsing.** Leveraging architectures like MS-TCN (83, 84), state-of-the-art models segment the continuous video stream into biologically meaningful units, providing the necessary temporal boundaries for phase-specific clinical analysis.
- **Phase 2: Quality Quantification (AQA Engine).** Unlike opaque end-to-end mapping, optimal strategies documented in the literature employ a hybrid approach that combines explicit biomechanical rules (e.g., ROM) with implicit manifold learning (13), translating motion features into quantitative metrics trusted by clinicians (85).
- **Phase 3: Pathological Attribution Diagnosis.** Transcending simple scoring, advanced paradigms perform multi-label classification (73) to identify specific compensatory patterns (e.g., shoulder hiking), converting mathematical features into explicit diagnostic narratives to inform downstream interventions (49).

### 4.3 The coaching domain: closed-loop guidance and motor relearning

Serving as the “exit” terminal of the pipeline, literature within the Coaching domain builds upon **Motor Learning Theory** (86), exploring how to construct a closed sensorimotor loop aimed at resolving the patient’s **“Adherence Dilemma.”**

- **Semantic Translation (From KR to KP):** Research in this area translates abstract features into actionable **Knowledge of Performance (KP)** signals (87), utilizing semantic mapping techniques to guide movement correction with high precision (88).
- **Adaptive Intervention Strategy:** To balance **Cognitive Load**, leading intervention protocols utilize a “Feedback Pyramid” based on the Guidance Hypothesis (39), dynamically adjusting feedback frequency to promote the internalization of motor skills (89).
- **Immersive Multimodal Interaction:** By integrating AR-based visualization (90) and auditory biofeedback (Sonification) (91), current applications provide multisensory cues that may support action observation, movement awareness, and sensorimotor engagement (92, 93).

### 4.4 Operational application of the PAC taxonomy: example-based classification and evaluation

To avoid treating the PAC taxonomy as a purely conceptual framework, this review further operationalizes it as a practical classification and evaluation tool. As shown in Table 4, vision-based rehabilitation systems can be classified according to whether they perform only movement capture, provide clinically meaningful movement-quality assessment, or close the loop through patient-facing feedback and adaptive coaching. This operational mapping helps distinguish pose-estimation backbones (33, 70, 72, 80–82) from rehabilitation assessment systems (32, 42, 60, 85, 94, 95) and from full closed-loop coaching platforms (63, 68, 87, 88, 93, 96).

**Table 4:** Operational use of the PAC taxonomy for classifying and evaluating vision-based rehabilitation systems, with representative evidence from the reviewed literature.

PAC operational class	Core function and PAC-based interpretation	Representative evidence
Perception-only system	Captures body landmarks, joint coordinates, 2D/3D pose, or skeletal trajectories from RGB/RGB-D video or markerless pose-estimation pipelines. These systems provide the visual measurement backbone, but do not yet generate rehabilitation-specific interpretation, clinical scoring, or patient-facing feedback.	Pose reconstruction and markerless sensing studies (33, 70, 72, 80–82); markerless motion-capture reviews (10, 12).
Perception–assessment system	Converts pose trajectories or kinematic features into rehabilitation-relevant indicators, such as ROM, movement smoothness, compensatory movement, gait parameters, or action quality scores. These systems bridge visual sensing and clinical interpretation, but usually do not close the therapeutic feedback loop.	Rehabilitation assessment and AQA studies (22, 32, 42, 60, 85, 94, 95).
Full PAC-loop system	Integrates movement capture, movement-quality assessment, and corrective guidance through visual feedback, auditory biofeedback, AR-based feedback, or adaptive exercise coaching. These systems link measurement, reasoning, and patient-facing intervention within a closed-loop rehabilitation process.	App-based feedback, telerehabilitation, AR feedback, and biofeedback systems (63, 68, 87–89, 92, 93, 96).
Emerging PAC-extension system	Uses MLLMs, generative AI, or agent-like architectures to integrate visual input, pose traces, patient context, and semantic reasoning for individualized explanation or coaching. These systems extend PAC toward semantic coaching, but require prospective clinical validation, safety control, and regulatory assessment.	LLM- and GenAI-related rehabilitation or medical-AI studies (27, 30, 35–38, 49, 97).

This table-based operationalization also clarifies how the PAC taxonomy can guide future system development. A clinically mature system should not only capture movement accurately, but should also provide clinically interpretable assessment, explicit error attribution, and safe feedback delivery within a realistic rehabilitation workflow. By contrast, systems that report only pose-estimation accuracy or benchmark-level performance should be interpreted as technical foundations rather than complete rehabilitation solutions (10, 12, 89, 97).

This operational use of the PAC taxonomy provides two benefits for evidence synthesis. First, it prevents overestimating the clinical maturity of systems that achieve high pose-estimation accuracy but do not report rehabilitation-specific metrics or clinical validation (10, 33, 80, 81). Second, it identifies the missing link in many current systems: although Perception and Assessment modules are increasingly mature, fewer studies complete the full loop from visual sensing to clinically grounded feedback and adaptive coaching (42, 49, 68, 85, 87, 88). Therefore, the PAC taxonomy can serve not only as a descriptive map of existing literature but also as a design checklist for future vision-based rehabilitation systems. A clinically mature system should ideally demonstrate robust perception, clinically valid assessment, interpretable error attribution, and safe feedback delivery within a realistic rehabilitation workflow (32, 60, 63, 68, 88, 95). This operationalization does not constitute formal clinical validation of the taxonomy, but it demonstrates how the PAC framework can be practically applied to classify existing systems, evaluate their clinical maturity, and guide the design of future rehabilitation solutions.

## 5 The perception domain

Functioning as the “eyes” of intelligent rehabilitation systems, the core mandate of research within the Perception domain transcends the mere pursuit of minimizing Mean Per Joint Position Error (MPJPE) benchmarks on standard datasets. Instead, the primary objective observed in recent literature is to construct high-fidelity digital twins endowed with **Biomechanical Consistency** within uncontrolled, in-the-wild home environments. To surmount the significant “Sim-to-Real” domain shift, current methodologies collectively form a robust four-stage pipeline.

### 5.1 Data acquisition and preprocessing: hardware evolution and environmental challenges

The selection of data acquisition terminals directly dictates the deployment cost and data quality of rehabilitation systems, reflecting the inevitable trend of technology shifting from “controlled lab settings” to “ubiquitous home access.”

- **Paradigm Migration: From Specialized Sensors to Ubiquitous Perception Hardware.** Early rehabilitation vision research relied heavily on RGB-D sensors based on Structured Light or Time-of-Flight (ToF) technologies (e.g., Microsoft Kinect, Azure Kinect). The depth channel provided an absolute **Metric Scale**, making measurements of stride length or reach distance direct and accurate. However, prohibitive hardware costs and limited detection ranges hindered their large-scale deployment in telemedicine. Currently, mainstream research is accelerating its convergence towards ubiquitous visual perception solutions based on consumer-grade RGB cameras (e.g., smartphones). The Motion Coach system by Biebl et al. (63) demonstrated the feasibility of using standard smartphones for real-time correction of six knee/hip exercises in home settings, while Yeung et al.’s SmartRehab (43) validated the consistency between ubiquitous RGB solutions and the gold-standard Kinect in stroke upper limb rehabilitation. Furthermore, Zhu et al. (98) demonstrated that through post-processing, gait assessment scores from mainstream visual algorithms (e.g., BlazePose (72)) achieved a correlation coefficient as high as 0.99 with Vicon, indicating that low-cost solutions possess significant potential for clinical measurement.
- **Technical Challenges Facing Ubiquitous Perception.** Despite their high **Accessibility**, comparative studies by Dill et al. (33) point out that the 3D reconstruction error of conventional vision-based frameworks (MPJPE ≈ 56mm) is significantly higher than that of multi-view fusion systems (≈ 30mm), primarily facing two major challenges:
  - *Scale Ambiguity:* Single-stream RGB signals **inherently lack** depth information. Resolving this typically requires combining the **Weak Perspective Projection** assumption or utilizing human height priors for **Anthropometric Scaling**. As shown in PLIKS by Shetty et al. (79), utilizing parametric models to recover absolute scale is a critical pathway documented in the literature.
  - *Viewpoint Dependence:* Experiments by Aguilar-Ortega et al. (70) on the UCO dataset indicate that general models are highly sensitive to viewpoints. For instance, assessing Knee Valgus requires a frontal view, whereas assessing Kyphosis requires a sagittal view. If algorithms lack automated viewpoint normalization capabilities, they significantly increase the patient’s operational **Cognitive Load**. Research by Pereira et al. (76) also highlighted the significant impact of viewpoint discrepancies on MediaPipe’s shoulder ROM measurement results.

The aforementioned Scale Ambiguity and Viewpoint Dependence fundamentally reflect the limitations of relying solely on image features. This serves as the starting point for the field’s emphasis on shifting from “Geometric Reconstruction” to the **“Biomechanical Digital Twin”** paradigm—external prior knowledge (such as anthropometric models) must be introduced to fill the missing depth information, thereby countering the “Projection Error” described in the *Clinical Context and Technical Challenges*.

**Figure 4:**
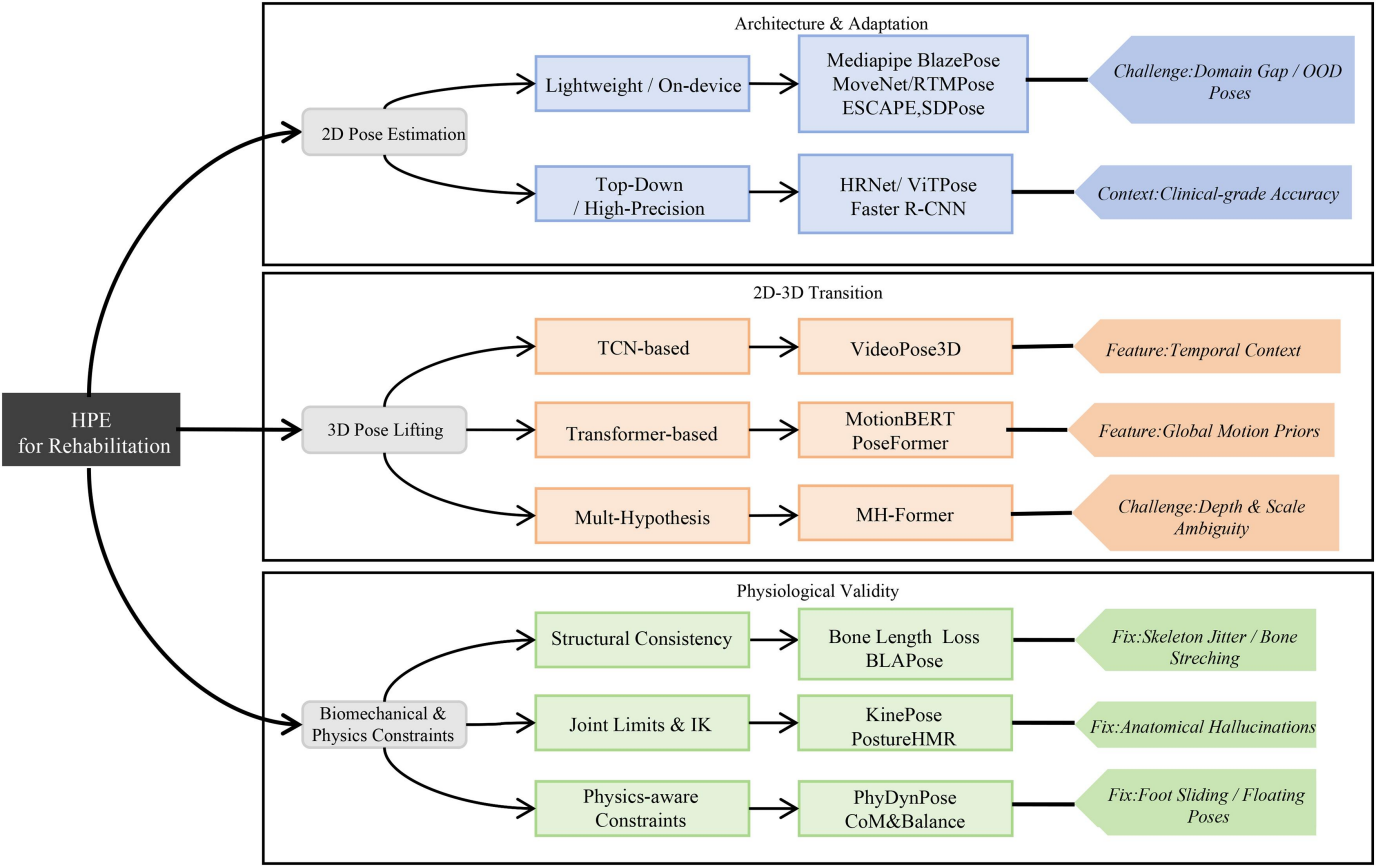
Taxonomy of human pose estimation algorithms in the perception domain. This hierarchical classification delineates the technical evolution path addressed in the *Perception Domain*, mapping specific algorithms to the engineering challenges they resolve. The roadmap is divided into three logical stages corresponding to the subsequent sections: *Architecture and Adaptation*, *2D-to-3D Transition*, and *Physiological Validity*.

To provide a structured roadmap for surmounting these challenges, a hierarchical taxonomy of Human Pose Estimation (HPE) algorithms for rehabilitation is illustrated in Figure 4. This classification serves as the structural foundation for the subsequent technical discussion, categorizing the literature into three critical stages: (1) **2D coordinate extraction and domain adaptation** (detailed in *2D Pose Estimation*); (2) **2D-to-3D pose lifting** (presented in *3D Pose Estimation and 2D-to-3D Lifting*); and (3) **the enforcement of physiological validity** through biomechanical and physics-aware constraints (formalized in *Physiological Validity*).

### 5.2 2D pose estimation: architecture selection and domain adaptation challenges

As the cornerstone of 3D reconstruction, the accuracy of 2D skeleton extraction directly dictates the performance ceiling of downstream applications. However, in rehabilitation scenarios, this process is plagued by a severe **“Sim-to-Real”** data gap.

- **Architectural Paradigms and Computational Trade-offs:**
  - **Top-down Approaches (e.g., HRNet):** These offer superior precision, making them ideal for precise single-person assessment. However, while Miao et al. (99) achieved excellent accuracy using a Faster R-CNN + HRNet combination in home settings, the computational cost becomes prohibitive in multi-person scenarios (e.g., when family members intervene).
  - **Lightweight On-device Models (e.g., MediaPipe BlazePose):** Designed specifically for mobile platforms, these utilize a hybrid strategy of regression heatmaps and coordinate offsets to achieve millisecond-level inference. To minimize latency, some studies propose extracting features directly from Motion Fields rather than reconstructing full frames, a shallow network design that significantly enhances convergence speed and energy efficiency (100). Simoes et al. (54) validated that MediaPipe achieves up to 99% classification accuracy for routine upper limb physical therapy exercises, making it the premier choice for home applications. However, as noted by Vineeth et al. (55), its stability in handling severe postural deviations or unconventional poses remains inferior to server-side large models.
- **The Domain Gap in Rehabilitation Scenarios:** This is the fundamental reason for the failure of general models in rehabilitation, primarily manifesting in the long-tailed effect of data distribution:
  - **Out-of-Distribution (OOD) Poses:** General datasets are dominated by upright actions. The UCO dataset (70) clearly demonstrated that supine movements cause a **“precipitous performance drop”** in general models unless specific rotation augmentation is applied. Addressing this, Bidulka et al.’s ESCAPE method (45) proposed an OOD detection and adaptive correction mechanism based on energy functions, while Liang et al.’s SDPose (50) utilized diffusion priors to enhance robustness across cross-domain data. These methods offer novel pathways for resolving the long-tailed distribution problem.
  - **Environmental Occlusion and “Ghost Limb” Misdetection:** Wheelchair armrests or walkers in home environments are frequently misclassified by models as “extra arms,” leading to skeletal collapse. Studies on UCO (70) and by Pornpipatsakul (53) have both emphasized the severity of this issue in pathological gait analysis of the lower limbs, particularly regarding complex self-occlusion and false detections caused by assistive devices.

The aforementioned occlusion and domain gap issues in 2D pose estimation fundamentally stem from the absence of spatial depth information and biomechanical prior knowledge. Relying solely on pixel-level optimization has proven insufficient. This necessitates an urgent transition to the discussion on *3D Pose Estimation and 2D-to-3D Lifting*, where recent studies fundamentally resolve these ambiguities and uncertainties in 3D space by introducing **Temporal Lifting** technology and **Biomechanical Constraints**.

### 5.3 3D pose estimation and 2D-to-3D lifting

Core clinical assessment metrics (e.g., Range of Motion, ROM) are inherently 3D spatial parameters. The perspective **Foreshortening** effect in 2D projections leads to severe measurement errors. Consequently, recovering 3D posture from 2D video (**3D Pose Lifting**) represents a critical leap “from image to data.”

#### 5.3.1 Evolution of solving the ill-posed problem

Recovering 3D from 2D is mathematically an **Ill-posed Problem**. Technological evolution has undergone a qualitative leap from “single-frame regression” to “temporal smoothing”:

- **Temporal Convolutional Networks (TCN):** VideoPose3D by Pavllo et al. (80) leverages temporal context from adjacent frames to smooth depth predictions. By using dilated convolutions to expand the receptive field, it effectively suppresses the **Jitter** inherent in single-frame predictions through motion continuity.
- **Transformers with Global Attention:** PoseFormer by Zheng et al. (101) demonstrated the superiority of Transformers in capturing long-range spatiotemporal dependencies. For actions with specific rhythms, such as Parkinsonian gait, this global attention mechanism more effectively **imputes** spatiotemporal information lost due to occlusion.
- **Multi-Hypothesis Reasoning:** Addressing depth uncertainty in mainstream single-view frameworks, MH-Former by Li et al. (81) generates multiple plausible 3D pose hypotheses and fuses them via weighting. This effectively mitigates depth ambiguity and significantly enhances reconstruction accuracy under non-standard viewpoints.

#### 5.3.2 Current state of clinical accuracy

Comparative studies using “gold-standard” optical motion capture systems, such as Vicon or OptiTrack, indicate that the Mean Per Joint Position Error (MPJPE) of state-of-the-art visual 3D pose estimation models in standard gait-analysis settings has been reduced to approximately 30–50 mm (33, 98). The HGcnMLP model by Hu et al. (64) reported knee joint angle measurements that were highly consistent with Vicon-based assessment in musculoskeletal gait analysis, with ICC values of 0.84–0.98 and angular errors of approximately $5^{\circ }-10^{\circ }$. These findings suggest that vision-based pose estimation has potential utility for monitoring large-joint movements such as squats and gait. However, such agreement should not be interpreted as sufficient evidence of clinical validity across all rehabilitation populations, movement tasks, camera viewpoints, or home environments.

Importantly, MPJPE is an engineering accuracy metric rather than a direct clinical validity endpoint. A low MPJPE value on standard datasets does not necessarily guarantee accurate ROM estimation, reliable compensatory-movement detection, agreement with therapist ratings, sensitivity to pathological movement patterns, or robustness in home-based rehabilitation. Therefore, clinical interpretation requires additional validation against biomechanical or clinical reference standards, such as optical motion capture, IMUs, depth-camera systems, goniometry, clinical scales, therapist ratings, or expert annotations.

To clarify this distinction, Table 6 is intended as a technical paradigm table that summarizes the evolution from data-driven pose regression to biomechanically constrained digital-twin modeling, rather than as direct evidence that MPJPE improvements alone establish clinical effectiveness. To further address clinical validity, we added Table 5, which summarizes explicitly coded validation approaches, reference standards, and comparator availability identified within the Perception-domain subset. This table distinguishes records validated against clinical or biomechanical reference standards from records reporting only benchmark-level pose-estimation metrics or lacking an explicitly coded clinical comparator. Because the table is based on conservative record-level coding, low counts in some categories should be interpreted as evidence of limited explicitly reported validation within the coded Perception-domain subset, rather than as evidence that such technologies are absent from the broader rehabilitation literature. The detailed record-level appraisal for all 147 included publications is provided in Supplementary Data Sheet 1, Table S2.

**Table 5:** Summary of validation approaches, reference standards, and comparator availability relevant to the perception domain.

Validation category	Reference standard/comparator	No. of coded records	Typical reported metrics	Clinical interpretation
Optical motion-capture validation	Vicon, OptiTrack, or laboratory-grade motion-capture systems	2	MPJPE, joint angle error, RMSE, ICC, and correlation with motion-capture trajectories	Optical motion capture represents the strongest biomechanical reference for validating 3D pose, joint kinematics, gait parameters, and ROM. However, only a small number of Perception-domain records were coded as using laboratory-grade optical motion-capture validation, indicating that many vision-based rehabilitation studies still lack direct validation against gold-standard biomechanical reference systems.
IMU or wearable-sensor comparison	Inertial measurement units, wearable motion sensors, or hybrid sensor systems	3	Joint angle error, gait parameters, temporal stability, RMSE, and correlation coefficients	IMU or wearable-sensor comparison provides a pragmatic validation route for ambulatory and home-based assessment. However, such systems are not always equivalent to optical motion capture for full-body biomechanical validation.
Depth-camera or RGB-D validation	Kinect, Azure Kinect, RGB-D cameras, or depth-assisted systems	1	Skeleton tracking accuracy, ROM, gait parameters, ICC, and agreement with reference depth systems	Depth-camera and RGB-D systems provide clinically accessible and relatively low-cost validation routes. However, the low number of explicitly coded records suggests that depth-assisted validation was less frequently reported in the Perception-domain subset, and sensor-specific performance may not generalize to monocular RGB or smartphone-based rehabilitation systems.
Clinical metric or clinical-scale reporting	ROM assessment, clinical scores, gait parameters, movement smoothness, or other clinically interpretable indicators	13	ROM error, clinical score agreement, gait parameters, ICC, RMSE, and sensitivity to functional status	Clinical metric reporting helps bridge engineering outputs with rehabilitation-relevant outcomes. Nevertheless, metric reporting alone does not necessarily imply validation against a gold-standard comparator or sufficient evidence of clinical effectiveness.
Therapist rating or expert annotation	Physiotherapist ratings, expert labels, clinical annotations, or movement-quality labels	1	Classification accuracy, action-quality score, agreement with expert labels, and compensatory-movement detection	Therapist ratings and expert annotations are important for translating perception outputs into clinically meaningful assessment, especially for movement-quality interpretation and compensatory-pattern recognition. The low number of explicitly coded records indicates that expert-annotated validation remains underreported within the Perception-domain subset.
Benchmark-only validation	Human3.6M, COCO, MPI-INF-3DHP, UCO, or other public datasets without direct clinical comparator	8	MPJPE, PCK, mAP, 2D/3D keypoint accuracy, and frame-level accuracy	Benchmark-only validation is useful for engineering comparison and algorithm development. However, it is insufficient by itself to establish clinical validity, safety, or generalizability to pathological movement in home-based rehabilitation.
No explicitly coded clinical comparator	Prototype, conceptual, preprint, technical, or contextual records without an explicitly coded reference standard in Supplementary Table S2	54	Feasibility description, qualitative demonstration, system architecture, conceptual evidence, or not specified in title/metadata	These records are useful for mapping emerging directions and technical background, but they should not be interpreted as direct clinical measurement validation unless comparator details are confirmed in the original full text.

The full record-level coding for all 147 included publications is provided in Supplementary Data Sheet 1, Table S2; the present table summarizes the subset most directly relevant to Perception-domain validation and comparator availability. Counts refer to Perception-domain records coded in Supplementary Data Sheet 1, Table S2, rather than mutually exclusive clinical trials or pooled effect-size units. Categories are not mutually exclusive because some records used more than one validation approach. Low counts in some comparator categories reflect conservative record-level coding and indicate limited explicitly reported validation within the Perception-domain subset, rather than the absence of related technologies in the broader rehabilitation literature. Clinical metric reporting was coded separately from gold-standard validation and should not be interpreted as direct clinical validation unless an external reference standard was reported. The category “No explicitly coded clinical comparator” indicates that no reference standard or clinical comparator was coded in Table S2; it does not necessarily mean that the original full text contained no validation information. Record counts should therefore be interpreted as evidence-mapping indicators rather than quantitative meta-analytic denominators. MPJPE: Mean Per Joint Position Error; ICC: Intraclass Correlation Coefficient; RMSE: Root Mean Square Error; ROM: Range of Motion; IMU: inertial measurement unit.

**Table 6:** Validation of perception domain: biomechanical constraints vs. pure data-driven paradigms.

Paradigm	Repres. Arch.	Comput. Logic	Biomech. Const.	MPJPE (mm)	Clinical metric	Inference cost	Data sources
Static spatial	MediaPipe, OpenPose, HRNet	Local pixel regression; ignores inter-frame.	**Implicit Stats** (Image features)	60–90	RMSE: $>10^{\circ }$ ICC: <0.75	**Minimal** (<30 ms)	(9, 12)
Temporal lifting	VideoPose3D, MotionBERT	1D dilated conv or RNN; context aggreg.	**Temporal Smoothness**	40–50	RMSE: $6^{\circ }-10^{\circ }$ ICC: 0.75–0.85	**Medium** (GPU)	(9, 102)
Kinematic informed	KinePose, BioHPE, HybrIK	IK and joint chain modeling.	**Hard Geom.** (DoF/Bone)	45–55	RMSE: $<5^{\circ }$ ICC: 0.85–0.95	**Med-High** (Embed.)	(9, 103)
Digital twin	SMPLify-X, PhysDynPose	Parametric Body Mesh; energy minimiz.	**Physical** (GRF; CoM; Coll.)	55–75	RMSE: $4^{\circ }-7^{\circ }$ ICC: >0.90	**Very High** (Server)	(9, 103)
Foundation model	PoseFormer, TokenPose	Global attention; Visual Token reasoning.	**Semantic Priors** (Consistency)	<45	n.a.	**Very High** (Cloud)	(9, 102)

1. **Eng. vs. Clin. Corr.:** Clinical metrics projected from pilot studies; MPJPE benchmarked on Human3.6M dataset.

2. **Rationale:** This table validates the necessity of introducing **Physical/Anatomical Priors** to eliminate “visual hallucinations” (ICC >0.85).

3. **Origin:** Data synthesized from core reviews (9, 12, 102, 103).

Taken together, current evidence suggests that the clinical readiness of perception systems depends not only on lower pose-estimation error, but also on anatomical consistency, clinically interpretable kinematic outputs, reference-standard validation, and robustness under real-world rehabilitation conditions. This provides the empirical rationale for the subsequent discussion of biomechanical constraints and digital-twin modeling.

### 5.4 Mathematical formalization: the biomechanical digital twin

To address the challenge of “visual hallucinations” documented in conventional systems, recent methodologies formalize 3D reconstruction as an **Energy Minimization Problem** based on parametric human models rather than simple regression tasks. Specifically, the Skinned Multi-Person Linear (SMPL) model (25, 26) serves as the mathematical foundation for Constructing biomechanical digital twins that decouple biological shape from movement posture. In these optimization-based paradigms (104), researchers typically optimize pose parameters θ (joint angles), shape parameters β (anthropometric characteristics), and global translation T by minimizing a composite loss function $E_{total}$ as shown in Equation 1:

$$\theta^{*},\beta^{*}={\arg\;\min}_{\theta ,\beta }\left(E_{\text{data}}+\lambda_{\text{anat}}E_{\text{anat}}+\lambda_{\text{temp}}E_{\text{temp}}+\lambda_{\text{\textbackslash{},phys}}E_{\text{\textbackslash{},phys}}\right)$$ (1)

Weighting factors $\lambda_{anat},\lambda_{temp},\lambda_{\;phys}$ are commonly determined through heuristic tuning or Bayesian optimization. Literature indicates that when analyzing pathological gait, increasing $\lambda_{anat}$ enforces anatomical consistency and prevents the models from overfitting to environmental noise. The core constraints identified in the literature are categorized as follows:

- $E_{data}$ (**Reprojection Consistency**): This term ensures visual alignment by minimizing the Euclidean distance between projected joints of the 3D model and the detected 2D keypoints.
- $E_{anat}$ (**Anatomical Prior**): These constraints enforce joint limits (e.g., knee extension $\leq 0^{\circ }$) and utilize learned prior distributions [e.g., VPoser (105)] to penalize postures outside the anthropometric manifold, thereby preventing skeletal collapse.
- $E_{\text{temp}}$ (**Structural Integrity**): By enforcing constant shape parameters β across video sequences, this term eliminates unnatural bone length variation. Additionally, minimizing the second derivative of pose ($\|\ddot{\theta }{\|}^{2}$) acts as a biomechanical low-pass filter to suppress high-frequency jitter.
- $E_{\;phys}$ (**Contact and Dynamics**): Recent studies utilize zero-velocity constraints on vertices detected in contact with the environment (e.g., foot-ground contact) to resolve “foot sliding” or “floating” artifacts.

#### 5.4.1 Structural consistency: ensuring rigid-body properties

Literature identifies bone length constancy as a primary requirement for clinical validity. Current strategies include:

- **Explicit Skeleton Constraints:** Approaches such as the Bone Length Consistency Loss (82) or post-processing templates like BLAPose (106) force models to maintain rigid-body properties.
- **Intrinsic Consistency via Parametric Models:** SMPL-based methods **inherently guarantee** temporal consistency by decoupling shape from pose, eradicating jitter caused by frame-by-frame independent predictions.

#### 5.4.2 Anatomical constraints: correcting joint violations

The baseline for clinical validity relies on adhering to anatomical joint limits.

- **Inverse Kinematics (IK) Optimization:** Paradigms like KinePose (48) model the body as a kinematic chain, utilizing IK to ensure degrees of freedom (DoF) and ROM constraints are satisfied.
- **Learned Pose Priors:** Advanced mesh-based methods (51) utilize probabilistic models (e.g., VPoser) to distinguish **“pathological anomalies”** from **“anatomical impossibilities,”** providing more robustness than hard-threshold IK.

#### 5.4.3 Physics-aware constraints: enforcing balance and contact

Integrating physics engines into pose estimation is the current frontier for addressing occlusion and floating issues.

- **Center of Mass (CoM) and Balance:** Research by Kim (107) introduces CoM constraints to adhere to static equilibrium conditions within the Base of Support (BoS).
- **Mesh-based Contact Modeling:** PhysDynPose (108) and similar physics-based optimizations utilize surface geometry to achieve precise Ground Reaction Force (GRF) calculation and **Self-collision Detection**, ensuring generated movements comply with Newtonian laws and provide a reliable basis for clinical fall risk assessment.

## 6 The assessment domain

Leveraging high-fidelity biomechanical digital twins, the primary mandate within the **Assessment Domain** is to transcend the **“Semantic Gap”** existing between low-level geometric representations and high-level clinical decision-making. As established in current literature, while general Human Action Recognition (HAR) focuses on identifying “action categories,” the clinical utility of rehabilitation relies on the **Quantification** of movement quality and the **Attribution** of pathological mechanisms. Consequently, advanced methodologies must surpass the constraints of *opaque feature mapping* inherent in traditional end-to-end models, establishing explainable reasoning pipelines aligned with the principles of **Evidence-Based Medicine (EBM)**. This section synthesizes existing technical paradigms following the methodological trajectory of “Temporal Semantic Parsing → Quality Quantification → Pathological Attribution.”

### 6.1 Pre-assessment modeling: movement segmentation and phase identification

The transition from raw data to clinical scoring requires a prior decoding of the movement’s temporal topology. Unlike the Perception domain, which prioritizes per-frame precision, research in this phase concentrates on parsing continuous motion into semantic units, thereby establishing the necessary temporal boundaries for phase-specific clinical analysis.

- **Methodological Strategies for Temporal Parsing:** Literature suggests that conventional threshold-based methods are often inadequate for handling high-frequency jitter in Parkinsonian or stroke-induced motor impairments. Consequently, state-of-the-art research frequently adopts **MS-TCN++** (Multi-Stage Temporal Convolutional Network) (83, 109). As elucidated by Filtjens et al. (84), MS-TCN++ utilizes a **Hierarchical Refinement Mechanism** to capture long-range temporal dependencies, enabling a robust decomposition of motion streams into semantically distinct sequences (e.g., Start → Descent → Hold → Ascent → End), even amidst kinematic noise.
- **Clinical Phase Identification:** The ability to distinguish muscle contraction types—specifically **eccentric vs. concentric** phases—is critical in post-operative scenarios such as ACL reconstruction. Studies by Averell (110) and Goldbraikh (111) demonstrate how such architectures precisely delineate phases with explicit biomechanical significance (e.g., “grasp-release”), thereby facilitating targeted, stage-specific clinical assessments.

This precise delineation effectively equips evaluation systems with a **“Biomechanical Clock.”** Algorithmically generated boundaries serve as **“Temporal Masks,”** addressing the inherent difficulty of locking onto correct assessment windows. This methodological rigor ensures that downstream analysis operates exclusively within valid movement phases, systematically eliminating interference from irrelevant data.

### 6.2 Computational paradigms for action quality assessment (AQA)

Research in Action Quality Assessment (AQA) seeks to map complex motor performances into quantifiable metrics. To overcome the lack of explicit decision-making evidence in early models, current literature is primarily bifurcated into two trajectories: **Explicit Rule-based** and **Implicit Learning-based** paradigms.

To clarify the applicable boundaries of these trajectories, Table 7 provides a taxonomic comparison of core logic, clinical suitability, and technical trade-offs. While explicit geometric methods offer superior interpretability, high-dimensional feature extraction becomes indispensable for addressing complex neurological features, such as pathological synergies.

**Table 7:** Taxonomy of computational paradigms in the assessment domain.

Paradigm	Rep. Models	Core logic	Clinical scenario	Pros	Cons	Sources
Explicit geometric	DTW, GMM, Template	Calculates similarity between trajectories and expert templates.	**Orthopedic:** Repetitive single-joint tasks.	No large training sets; high interpretability.	Sensitive to noise; no non-linear scaling.	(87, 112)
ST-graph (ST-GCN)	JR-GCN, EGCN++, ST-GCN	Models topological connections and dynamic evolution.	**Neurological:** Multi-joint synergy (e.g., gait).	Captures “compensatory coupling” features.	Gradient vanishing in long sequences.	(102, 113)
Fine-grained attention	HyperFormer, TPT, MixSTE	Learns weights for action phases via global attention.	**Functional:** Complex tasks (e.g., Fugl-Meyer).	SOTA accuracy; identifies subtle pathology.	Massive cost; data-heavy.	(60, 102)
Contrastive ranking	Co-Rehab, PCLN, Siamese Net	Learns relative distance between test and reference videos.	**Personalized:** Longitudinal progress tracking.	Resolves pathological sample scarcity.	Lacks explicit physiological metrics.	(86, 102)

**Synthesis Rationale:** This table establishes a taxonomic baseline, validating the adoption of a **“Hybrid Architecture”**—integrating explicit rules for orthopedic objective standards with implicit manifold learning for complex neurological synergies—as an optimal path to resolve the clinical semantic gap.

#### 6.2.1 Paradigm A: explicit geometric and rule-based methods

This paradigm utilizes geometric indicators derived from biomechanical definitions, providing robust **Clinical Interpretability**.

- **Evolutionary Trajectory:** Research by Sun et al. (87) utilized Dynamic Time Warping (DTW) for trajectory similarity, while Seredin et al. (88) optimized weight allocation via balanced time-warping. Furthermore, SR-POSE (114) demonstrated the feasibility of real-time assessment through lightweight geometric computation.
- **Clinical Suitability:** This approach aligns with orthopedic rehabilitation where standards are objective (e.g., TKA ROM targets). By providing explicit decision-making evidence, it remains the most trusted paradigm for clinical practitioners.

#### 6.2.2 Paradigm B: implicit learning and manifold modeling

This paradigm leverages deep networks to learn the high-dimensional **Manifold** of movements, capturing non-linear dynamic features difficult to define manually.

- **Graph and Attention Modeling:** To characterize human topology, Deb et al. (85) demonstrated that ST-GCN improves scoring accuracy by learning joints’ physical connections. Similarly, skeleton-based Transformers (73) have been explored to capture long-range dependencies in extensive motion sequences (>300 frames).
- **Anomaly Detection:** To mitigate pathological data scarcity, Reiss (115) and Cherian (116) utilized One-Class Classification to learn a “standard manifold” from correct movements, using distance metrics as a measure of quality.
- **Contrastive Learning:** Recent studies (42, 56) employ hierarchical contrastive learning to extract fine-grained features, enhancing discriminative power in label-scarce rehabilitation scenarios.

Table 8 provides a quantitative analysis validating the **“Hierarchical Fusion”** strategy. The synthesis indicates that compared to low-level regression (ρ≈0.41), the integration of **fine-grained temporal parsing** and **ensemble** methods elevates assessment consistency to expert-level standards (ρ>0.90).

**Table 8:** Validation of assessment domain: quantitative evidence of methodological evolution.

Ladder	Rep. Arch.	Context	Metrics	Result	Evolutionary analysis	Sources
1. Signal Regression	L-SVR	Surgery	Spearman ρ	0.41	Relies on shallow features; fails to identify compensations.	(102)
2. Spatio-temp.	C3D/LSTM	Surgery	Spearman ρ	0.65–0.72	Implements temporal modeling; suffers from boundary ambiguity.	(102)
3. Fine-grained	TPT	Surgery	Spearman ρ	**0.89–0.92**	Aligns implicit features with clinical logic via phase locking.	(102)
4. Expert Driven	HMM	Stroke	Frame Acc	77.82%	Reliant on expert matrices; limited home-based robustness.	(60)
5. Hierarch. Fusion	**Ensemble**	Stroke	Frame Acc	**85.08%**	**Optimal Paradigm:** Balances deep features with clinical rigor.	(60)
6. Clinical Alignment	Kinect	PT	Correl. (r)	0.65-0.90	Validates consumer sensors; lacks personalized adaptation.	(90)

Bold values indicate the best reported result within each metric category among the studies summarized in the table.

### 6.3 Pathological attribution reasoning and multi-label error localization

The clinical value of rehabilitation guidance lies in diagnosing the underlying causes of performance degradation (the “Why”). This necessitates an **Attribution Capability**—the ability to back-propagate from the feature space to specific pathological mechanisms.

- **Multi-label Compensation Detection:** Pathological motion often manifests as a hybrid of compensatory modes. Recent research (117) utilizes 3D reconstruction coupled with foundation models to achieve frame-level classification of stroke compensations. While some detection algorithms (94) originated from pressure data, their core philosophy—real-time multi-class identification—aligns with current vision-based objectives. Evidence (73) confirms that ST-GCN-based models identify error categories with higher precision than traditional methods, enabling joint-specific attribution.
  This phase acts as the **“Diagnostic Nexus”** of the Assessment Domain, bridging raw motion data with clinical reasoning. By transcending holistic regression, it enables pinpoint localization of **“micro-errors”** (e.g., subtle trunk lean), directly addressing the clinical requirement for compensatory monitoring.
- **Semantic Reasoning and Clinical Narratives:** To bridge the semantic gap, systems must translate abstract features into structured narratives. Tang et al. (49) demonstrated the use of explicit features (e.g., valgus angles) as an intermediate Domain to generate natural language reports. This signifies a shift from pure **numerical output** toward **semantic reasoning**, facilitating the integration of deep causal inference via Large Models (38).
- **Data Augmentation and Implementation:** Addressing the scarcity of error samples, **Error-Guided Pose Augmentation** (118) has been proposed to synthesize clinical error modes, improving classification for long-tailed distributions. On the implementation front, PosePilot (119) demonstrates how BiLSTM can be leveraged on **edge devices** for real-time rectification, providing a robust engineering reference for ubiquitous systems.

## 7 The coaching domain

Building upon high-fidelity perception and precision assessment paradigms, the **Coaching Domain** represents the pivotal interface for translating computational metrics into therapeutic actions. While the Perception and Assessment domains quantify motor states, the clinical value of rehabilitation technologies ultimately depends on whether these measurements can be converted into safe, interpretable, and patient-facing feedback. Current research guided by **Motor Learning Theory** explores how kinematic features can be transformed into biofeedback, augmented feedback, or adaptive coaching strategies to support motor relearning and adherence.

However, the Coaching domain should be interpreted more cautiously than the Perception and Assessment domains. Compared with pose-estimation accuracy studies or movement-quality assessment studies, direct clinical evidence for automated coaching remains relatively limited. Many existing works report feasibility, prototype-level feedback generation, case-series evidence, protocols, or evidence synthesized from secondary reviews rather than primary randomized or controlled clinical trials in rehabilitation populations. Therefore, this section synthesizes coaching strategies as promising and theory-informed approaches, while distinguishing them from definitively validated clinical interventions.

### 7.1 Content translation: the paradigm shift from knowledge of results (KR) to knowledge of performance (KP)

Effective rehabilitation feedback transcends simple error detection and ideally functions as a prescriptive conduit for motor correction. To address the cognitive barriers that prevent patients from rectifying recognized errors, the literature emphasizes a semantic transition from summative **Knowledge of Results (KR)** to process-oriented **Knowledge of Performance (KP)**.

- **Knowledge of Results (KR):** Conventional applications typically provide KR, namely summative and coarse-grained feedback such as binary completion status, repetition count, or overall score. In neurorehabilitation and complex orthopedic rehabilitation contexts, such feedback may be insufficient because it does not explain how the movement should be corrected.
- **Knowledge of Performance (KP):** KP provides information about the quality of the movement pattern itself, such as joint alignment, trunk compensation, trajectory deviation, excessive asymmetry, movement smoothness, or timing errors. In principle, KP is more compatible with rehabilitation needs because it can support error attribution and corrective learning rather than only reporting task success or failure.

Although KP-based feedback is theoretically more informative than KR-only feedback for complex rehabilitation tasks, direct controlled evidence comparing KR-only and KP-based feedback in vision-based rehabilitation remains limited. Therefore, claims regarding the superiority of KP should be interpreted as consistent with motor-learning theory and preliminary rehabilitation evidence rather than as definitive clinical proof. Future studies should directly compare KR-only feedback, KP-based corrective feedback, therapist-guided feedback, and adaptive multimodal coaching in randomized or controlled rehabilitation trials.

#### 7.1.1 Strategies for semantic translation

Current literature identifies several strategies for bridging the gap between raw data and actionable feedback:

- *Semantic Mapping Techniques:* Studies by Sun et al. (87) and Tang et al. (49) explore the conversion of explicit geometric features, such as knee angles or movement-quality scores, into natural-language corrective cues. This **Semantic Translation** may help bridge the gap between quantitative metrics and clinically understandable instructions. However, these systems should currently be interpreted as prototype-level or emerging evidence unless validated in prospective clinical trials.
- *Mitigating the Adherence Paradox:* By translating abstract kinematic parameters into intuitive instructions, coaching systems may reduce cognitive load and improve the usability of home-based rehabilitation. Nevertheless, the extent to which such feedback improves long-term adherence, functional recovery, or patient-reported outcomes remains insufficiently established.
- *Personalized Instruction Archetypes:* For specialized populations such as pediatric patients, paradigms like the REHAB-PAL system (120) use socially assistive robotics to translate training goals into personalized and child-friendly instructions. Such work highlights the importance of tailoring coaching style to patient age, cognitive capacity, and motivational needs, but additional rehabilitation-specific clinical validation is still required.

#### 7.1.2 Prioritization logic: the hierarchical feedback pyramid

To prevent cognitive overload, contemporary research often favors prioritized feedback rather than simultaneous reporting of all detected errors. This logic can be conceptualized as a **Feedback Pyramid**:

- **Phase 1—Safety-Critical Intervention:** High-priority alerts for hazardous movements, such as joint hyperextension, excessive trunk compensation, or unstable balance, should have the highest interrupt priority to prevent secondary injury.
- **Phase 2—Primary Metric Feedback:** Corrections centered on core rehabilitation goals, such as target ROM, weight-bearing symmetry, gait timing, or compensation reduction, should constitute the essential instructional content.
- **Phase 3—Optimization and Fine-tuning:** Subtle cues related to movement smoothness, rhythm, or efficiency should be provided only after safety and primary task criteria are satisfied.

This hierarchical logic is clinically plausible because it reflects how therapists often prioritize safety, task success, and movement quality. However, the optimal ordering, timing, and personalization of automated feedback priorities remain open empirical questions.

### 7.2 Intervention strategy and timing: regulation of cognitive load

In motor learning, the frequency and timing of feedback are critical. According to the **Guidance Hypothesis**, excessive feedback frequency may increase cognitive load and promote reliance on external cues, potentially impeding the development of endogenous proprioceptive control.

- **Concurrent Feedback paradigms:** These involve real-time corrections during movement execution. Concurrent feedback may be useful during early motor acquisition, safety-critical training, or relatively straightforward orthopedic exercises, but it may also increase dependency if used excessively (68, 89, 121).
- **Faded and Terminal Feedback strategies:** Research by Aoyagi et al. (39) suggests that a **faded feedback** approach, in which feedback frequency is gradually reduced as performance improves, can support motor learning retention. This principle is compatible with adaptive rehabilitation systems that progressively shift responsibility from external guidance to patient self-monitoring.

Nevertheless, most evidence on feedback scheduling comes from motor-learning experiments, rehabilitation protocols, or broader real-time feedback reviews rather than from large randomized trials of vision-based rehabilitation coaching. Future systems should therefore report not only short-term performance improvement but also retention, transfer, adherence, safety, and patient-reported usability.

### 7.3 Multimodal augmentation: immersive interaction and motor-learning support

To compensate for impaired proprioception, visual feedback can be supplemented with auditory, haptic, or immersive signals. These multimodal strategies are theoretically consistent with sensorimotor learning and action-observation mechanisms, but direct evidence that they support motor relearning in vision-based rehabilitation remains limited. Therefore, the term **neuroplasticity-oriented** is used here to describe a theoretical rehabilitation rationale rather than a confirmed mechanistic outcome.

- **Visual Augmentation via AR/VR:** Paradigms using HoloLens 2 or other AR/VR systems can project virtual skeletons, movement trajectories, or task goals to provide a **digital mirror** effect (93). Such visualization may help patients perceive trajectory deviations and compensate for reduced proprioceptive feedback. However, additional controlled studies are needed to determine whether AR/VR feedback improves functional outcomes beyond conventional therapist-guided feedback.
- **Auditory Biofeedback and Sonification:** Standardized parameter-to-sound mapping converts kinematic variables into auditory signals. Trials by Owaki et al. (96) suggest that auditory biofeedback can improve selected gait-control parameters in stroke rehabilitation. Reviews on sound-movement coupling also support the theoretical plausibility of sonification for timing and rhythm regulation (91). Nevertheless, the generalizability of these findings to camera-based home rehabilitation requires further validation.
- **Gamification and Immersive Engagement:** Recent studies and reviews (92, 122–124) suggest that gamified or immersive environments may improve motivation and engagement by transforming repetitive exercises into goal-oriented tasks. However, evidence for sustained adherence, reduced attrition, or improved patient-reported outcomes remains heterogeneous and should not be assumed from short-term usability or engagement measures alone.

The practical value of coaching also depends on real-time system performance. Literature such as Hribernik et al. (89) emphasizes that real-time feedback systems are constrained by **end-to-end latency**. Excessive delay may disrupt sensorimotor integration and reduce the usefulness of corrective feedback. Therefore, latency, stability, interpretability, and safety thresholds should be reported alongside clinical and usability outcomes in future coaching studies.

### 7.4 Evidence maturity and clinical interpretation of coaching strategies

Table 9 summarizes major coaching strategies according to their technical modality, theoretical basis, evidence maturity, and clinical interpretation. The table intentionally distinguishes primary clinical evidence from secondary reviews, protocols, prototype studies, and emerging AI-based feedback systems. This distinction is important because the current evidence base does not yet support treating all coaching strategies as equally validated clinical interventions.

**Table 9:** Synthesis of coaching strategies, evidence maturity, and clinical interpretation in vision-based rehabilitation.

Core logic	Technical modality	Theoretical basis	Evidence maturity and clinical interpretation	Sources
Semantic translation	Natural-language corrective feedback	KP-oriented prescriptive feedback and cognitive-load reduction	Prototype and emerging evidence. KP-based feedback may be more informative than KR-only feedback for complex tasks, but direct controlled evidence in vision-based rehabilitation remains limited.	(49, 87)
Intervention frequency	Faded or adaptive feedback scheduling	Guidance Hypothesis and motor-skill internalization	Theory-informed and partially supported by motor-learning evidence. Faded feedback may support retention, but large rehabilitation-specific trials using vision-based systems remain limited.	(39, 40, 89)
Latency management	Real-time monitoring and closed-loop feedback	Sensorimotor synchronization and feedback usability	Mainly technical and review-level evidence. Latency should be reported as a safety and usability constraint, but clinical thresholds require task- and population-specific validation.	(89)
Visual augmentation	AR/VR mirroring and trajectory visualization	Action observation and augmented visual feedback	Promising but heterogeneous evidence. AR/VR may support engagement and movement awareness, but direct evidence for functional superiority over conventional therapy remains limited.	(92, 93, 122–124)
Rhythmic entrainment	Auditory biofeedback and sonification	Auditory–motor coupling and temporal cueing	Includes primary clinical evidence in stroke gait rehabilitation, but generalization to camera-based home rehabilitation requires further validation.	(91, 96)
Active engagement	Gamified or socially assistive interaction	Motivation, adherence support, and patient-centered interaction	Feasibility and engagement-oriented evidence. Effects on long-term adherence, PROs, and functional recovery remain insufficiently established.	(92, 120, 122)

The evidence summarized in this table should be interpreted as preliminary and theory-informed rather than definitive clinical proof. Primary randomized or controlled clinical trials directly comparing KR-only feedback, KP-based corrective feedback, therapist-guided feedback, and multimodal adaptive coaching in vision-based rehabilitation remain limited.

KP, knowledge of performance; KR, knowledge of results; PROs, patient-reported outcomes.

Overall, the Coaching domain remains a critical but comparatively under-validated component of vision-based rehabilitation. Existing studies support the plausibility of semantic feedback, adaptive scheduling, sonification, AR/VR visualization, and gamified interaction, but the direct clinical evidence base remains less mature than that for movement capture and movement-quality assessment. Future research should prioritize randomized or controlled clinical studies that evaluate not only execution accuracy and kinematic improvement, but also retention, transfer to daily activities, safety, adherence, pain, satisfaction, quality of life, and functional independence. Such outcomes are essential for determining whether computationally sophisticated coaching systems translate into meaningful patient-centered rehabilitation benefits.

## 8 Discussion

The systematic analysis of existing literature reveals that vision-based rehabilitation is entering a transformative era. To fully realize the clinical utility of these technologies, we must examine both the evolutionary pathway of emerging computing paradigms and the persistent challenges blocking their practical deployment. This section discusses the future prospects of multimodal foundation models in rehabilitation alongside the clinical implementation, data ecology, regulatory, and ethical barriers that must be addressed to transition from academic prototypes to certified medical devices and routine clinical workflows.

### 8.1 Future prospects: reshaping rehabilitation computing via multimodal foundation models

The GenAI- and MLLM-related discussion in this section should be interpreted as an emerging and prospective research direction rather than as evidence of established clinical effectiveness. Current applications of foundation models in vision-based rehabilitation remain insufficiently validated in prospective rehabilitation trials. Therefore, claims regarding autonomous rehabilitation agents, generative visual feedback, Visual Self-Modeling, and MLLM-assisted coaching are framed as future hypotheses that require prospective clinical validation, safety evaluation, bias assessment, usability testing, and regulatory review before routine clinical deployment.

The paradigm of vision-based rehabilitation may be reshaped by the future integration of Multimodal Large Language Models (MLLMs) and Generative AI. Recent medical AI research suggests that foundation models may contribute to clinical decision support and interpretability, but their direct clinical utility in rehabilitation remains insufficiently validated. In this emerging and prospective paradigm, MLLMs may serve as a potential “Cognitive Hub” to support the transition from discriminative analysis to generative, patient-facing assistance. We therefore outline below a theoretical architecture of prospective autonomous rehabilitation agents, while emphasizing that these systems remain at an early translational stage.

To illustrate the theoretical necessity of this shift, we analyze the post-stroke movement **“Hand-to-Opposite-Shoulder”** as a conceptual archetype. This movement, involving complex joint decoupling and **Pathological Synergy**, serves as a suitable litmus test for evaluating the transition from geometric regression to semantic reasoning.

#### 8.1.1 Reshaping perception: from geometric reconstruction to semantic intuition

Traditional rehabilitation AI has historically been confined to **Geometric Reconstruction**, outputting coordinates that often fail to capture the **Functional Impairments** underlying motor deficits (125, 126). In the era of foundation models, perception is evolving toward **Intent-based Semantic Sensing**. Leveraging the long-horizon temporal reasoning of modern MLLMs (127), future systems may be able to assist in estimating motor intent and characterizing the dynamic gap between intended and observed movement, although this capability remains to be validated in prospective rehabilitation studies.

For the “Hand-to-Opposite-Shoulder” task (59), the literature suggests a shift toward **Zero-shot Parameter Estimation**, where systems extract mission-critical features directly from RGB video based on semantic instructions. This allows for the **Semantic Tagging** of obstacles, identifying high-level functional barriers such as [Elbow Flexion Dominance] or [Ipsilateral Trunk Lean] that transcend simple joint angles (126).

To reconcile the tension between data scarcity and privacy mandates, future frameworks should adopt **Federated Learning** and **Privacy-Preserving Analytics** (e.g., GDPR/HIPAA-compliant pipelines), enabling collaborative training across institutional boundaries without raw data exposure.

#### 8.1.2 Reshaping assessment: causal attribution of intent-reality conflict

The critical frontier in assessment is the transition from opaque scoring to a **“Diagnostic Nexus”** powered by **Chain-of-Thought (CoT)** reasoning. This paradigm focuses on resolving the **Intent-Reality Conflict**—the gap between a patient’s motor intent and their actual execution—by explaining why a movement fails to meet clinical targets through transparent diagnostic chains (52, 128). By articulating the underlying clinical logic, these frameworks provide physiotherapists with actionable insights that align with established *Evidence-Based Medicine (EBM)* pathways.

Recent research trends suggest that future frameworks utilizing CoT can theoretically map perceived visual phenomena to underlying neural control deficits:

- **Intent Analysis:** Decoupling joint control to assess the **Loss of Joint Individuation** typically seen in stroke survivors (129).
- **Mechanism Mapping:** Attributing involuntary elbow flexion to the activation of **Abnormal Flexor Synergy** caused by the inability of damaged corticospinal tracts to inhibit secondary torques (47, 95).
- **Diagnostic Synthesis:** Identifying compensatory strategies, such as shoulder hiking, used to shorten anatomical distances during failed reach tasks (130).

#### 8.1.3 Reshaping guidance: towards generative sensorimotor loops

The evolution of the Coaching domain involves upgrading interaction from mere “error reporting” to **“motor-learning support”** within a closed sensorimotor loop.

##### 8.1.3.1 Intelligent Decision-Making and Metaphorical Cueing

By leveraging MLLMs, future agents can generate instructions using an **“External Focus”** strategy, which has been proven to optimize motor performance (40). Examples observed in pilot studies include the use of metaphorical cues (e.g., “pulling a seatbelt”) to promote automated motor control and reduce cognitive load.

##### 8.1.3.2 Generative Visual Biofeedback: Visual Self-Modeling (VSM)

A potentially transformative application of Generative AI is **Visual Self-Modeling (VSM)** enabled by Text-to-Video (T2V) models (36, 131).

- **Paradigm Shift:** Moving beyond traditional mirror boxes, generative models can synthesize an **“Expected Recoverable State”** video of the patient successfully performing a movement with *suppressed synergy*. This visualization may support action observation, movement imagery, and self-efficacy, although direct evidence for mirror-neuron activation or neuroplastic change in this specific GenAI-based rehabilitation context remains limited.
- **Physical Realism:** To ensure clinical safety, future generative loops must integrate the biomechanical constraints discussed in the Perception domain, ensuring that AI-generated feedback remains anatomically authentic and physically inductive.

Ultimately, these advancements signify the emergence of **Autonomous Rehab Agents** capable of **Dynamic Prescription Regulation**, autonomously adjusting complexity and motivational content to support neuroplasticity-oriented motor relearning.

### 8.2 Barriers to clinical translation: clinical implementation, data ecology, and ethical safety

While the proposed framework illustrates a clear evolutionary path for vision-based rehabilitation, transitioning these academic prototypes into approved **Software as a Medical Device (SaMD)** remains a formidable challenge. The primary hurdle lies in shifting the paradigm from “algorithmic accuracy” to “clinical fidelity.” We dissect below the root challenges frequently overlooked in current literature and discuss potential pathways for large-scale clinical adoption.

#### 8.2.1 Clinical implementation and workflow integration

Beyond algorithmic accuracy, the transition of vision-based rehabilitation systems from experimental prototypes to routine clinical practice requires a broader implementation pathway. A first unresolved challenge is the lack of multicenter validation. Many existing systems have been evaluated in single-center, small-sample, or laboratory-controlled settings, whereas real-world deployment requires robustness across hospitals, rehabilitation protocols, therapist practices, camera positions, lighting conditions, home environments, and patient demographics (12, 33, 68, 132). Without such external validation, performance reported on controlled datasets may not translate into reliable clinical use.

A second challenge concerns generalization across pathological populations. Movement abnormalities differ substantially among stroke, Parkinson’s disease, osteoarthritis, sarcopenia, postoperative orthopedic rehabilitation, cerebral palsy, and low back pain populations. Models trained on healthy participants or mimicked impairments may fail to recognize disease-specific features such as abnormal synergy, tremor, spasticity, compensatory trunk movement, pain-avoidance strategies, or fatigue-related movement degradation (31, 60, 68, 94, 95, 129). Therefore, future studies should move beyond generic pose-estimation accuracy and evaluate whether computer vision outputs remain clinically meaningful across disease phenotypes, severity levels, and rehabilitation stages.

Integration into clinical workflows remains a practical bottleneck. For physiotherapists and rehabilitation physicians, an algorithmic score is insufficient unless it can be translated into clinically actionable information, such as the affected joint, movement phase, compensatory pattern, severity level, recommended correction, and longitudinal progress. Effective deployment therefore requires therapist-facing interfaces, electronic documentation compatibility, shared decision-making support, exercise prescription adjustment, and follow-up monitoring. Systems should reduce rather than increase clinician workload, particularly in settings already facing rehabilitation workforce shortages and limited access to therapy services (4, 5, 7, 24, 63). From the perspective of the PAC taxonomy, successful clinical translation requires not only robust Perception, but also clinically validated Assessment and safe, workflow-compatible Coaching.

#### 8.2.2 Privacy, consent, and data protection in home video rehabilitation

Privacy and data protection represent critical barriers for camera-based rehabilitation. Unlike wearable sensors that primarily record inertial signals, vision-based systems may capture identifiable facial information, body shape, caregivers, household environments, and other sensitive contextual data. Even skeletonized trajectories may retain re-identification risks when combined with temporal movement patterns or demographic information. Privacy-preserving video processing, on-device inference, data minimization, secure transmission, anonymization, and transparent consent procedures should therefore be treated as core design requirements rather than optional technical features (24, 46, 97).

For home-based video rehabilitation, consent procedures should be more specific than those used for conventional clinical data collection. Participants should be informed whether the system performs continuous or repeated video capture, whether raw video, skeleton trajectories, or derived kinematic features will be stored, how long data will be retained, whether data may be reused for algorithm development, and how patients can withdraw consent or request data deletion. In addition, because home-based cameras may inadvertently capture caregivers, family members, or household environments, future systems should implement bystander-protection mechanisms, privacy zones, automatic face or background blurring, and clear policies for secondary data use.

#### 8.2.3 Regulatory readiness and SaMD certification

Regulatory readiness is equally important for clinical translation. Systems that only estimate pose may function as low-risk measurement tools, but systems that generate clinical interpretations, detect abnormal movement patterns, recommend exercise modifications, or deliver automated coaching may approach the functional scope of Software as a Medical Device (SaMD). Such systems require evidence of analytical validity, clinical validity, usability, risk control, explainability, and post-deployment monitoring. This is particularly important for MLLM- or agent-assisted rehabilitation systems, which may generate fluent but unsafe exercise advice if hallucination control, uncertainty estimation, and clinician escalation mechanisms are not implemented (35, 37, 38, 49, 97).

More specific regulatory frameworks should also be considered. In the European Union, systems that provide clinical assessment, therapeutic recommendations, or adaptive exercise guidance may fall within the scope of the Medical Device Regulation (MDR 2017/745), and AI-enabled rehabilitation systems may also need to consider requirements related to the EU AI Act. In the United States, such systems may be evaluated within FDA digital health and Software as a Medical Device pathways. Accordingly, future vision-based rehabilitation systems should provide evidence for clinical performance evaluation, risk management, human factors and usability testing, cybersecurity, post-market surveillance, and change-management plans for adaptive or continuously updated algorithms. In addition to regulatory pathways, future systems should align, where applicable, with relevant medical-device software, safety, and usability standards, such as ISO 14971 for risk management, IEC 62304 for medical device software life-cycle processes, and IEC 62366-1 for usability engineering, depending on the intended use, risk classification, deployment setting, and jurisdiction.

#### 8.2.4 Data ecology: from mimicked data to pathological digital phenotypes

The rehabilitative computer vision domain currently faces a critical state of **“Data Anemia”** (112, 133). As synthesized in Table 10, a systematic survey of 21 mainstream datasets reveals a severe structural imbalance:

- **The Mimicry Gap:** The vast majority of available resources (Groups I & II, e.g., Human3.6M, UI-PRMD) rely on **Healthy Subjects**. Even those designed for rehabilitation typically utilize “mimicked” impairments. Such performance-based data tend to be kinematically over-standardized, failing to capture the non-linear, involuntary features like *flexor synergy* in stroke survivors or *pathological tremors* in Parkinson’s patients.
- **The Scarcity of Reality:** As shown in Group III of Table 10, datasets containing **Real Pathological Data** are exceptionally rare and small-scale (typically n<50). Furthermore, due to stringent privacy and ethical regulations, these resources are predominantly **Private** or **Restricted**, hindering the generalizability of deep learning models.

This distribution shift often leads to **“Simulation Hallucinations,”** where models trained on ideal data misinterpret pathological gait deviations as noise and erroneously “smooth” them out during clinical inference.

**Table 10:** Comprehensive survey of 21 vision-based datasets: from general benchmarks to pathological realities.

Dataset	Ref	Year	Task domain	Subj.	Modality	Subject type (bias source)
***I. General & Pre-training Benchmarks (Upright Prior Source)***						
Human3.6M	(34)	2014	Daily Activities	11	RGB, MoCap	Healthy (Prof. Actors)
COCO	(137)	2014	Object/Keypoint	>200k	RGB (2D)	General Public
Kinetics-400	(138)	2017	Sports/Events	N/A	RGB (Web)	Healthy (YouTube)
MPI-INF-3DHP	(139)	2017	General Motion	8	RGB (Multi)	Healthy
AMASS	(140)	2019	MoCap Archive	300+	MoCap	Healthy
NTU RGB+D	(141)	2019	Daily Actions	106	RGB-D	Healthy
***II. Simulated Rehabilitation & Fitness (The Mimicry Gap)***						
UI-PRMD	(112)	2018	Standardized PT	10	RGB-D, IMU	Healthy (Mimicking)
AHA-3D	(142)	2018	Senior Fitness	21	RGB-D	Healthy (Elderly Sim.)
Fitness-AQA	(143)	2019	Gym Exercises	N/A	RGB	Healthy (Expert/Novice)
KIMORE	(32)	2019	Low Back Rehab	78	RGB-D	Healthy + Mild Pain
Home Rehab	(132)	2020	Rehab Comparison	25	RGB-D, IMU	Healthy
Bridging-Ex	(53)	2023	Stroke Bridging	10	RGB	Healthy (Mimicking)
UCO-Rehab	(70)	2023	Rehab (Supine)	27	RGB	Healthy
Barbell-DEC	(144)	2024	Repetition Count	N/A	RGB	Healthy
***III. Proxy & Real Pathological Data (Data Anemia)***						
JIGSAWS	(145)	2014	Robotic Surgery	8	Stereo Video	Robotic Proxy (Fine-grained)
SPHERE	(146)	2016	Home Monitoring	∼50	RGB-D, IMU	Real Residents (Home Setting)
Hand-Tremor	(147)	2018	Tremor Freq.	N/A	Video	Real Patients
SARAH	(60)	2021	Post-Stroke Task	N/A	RGB	Real Patients (Stroke)
StrokeRehab	(57)	2022	Functional Reach	39	RGB	Real Patients (Stroke)
KGS (Knee)	(64)	2023	Knee OA Gait	80	RGB, MoCap	Real Patients (OA)
PD-Gait	(31)	2024	Parkinson’s Gait	N/A	RGB	Real Patients (Parkinson’s)
TUG-Sarcopenia	(68)	2024	Timed Up and Go	414	RGB	Real Patients (Sarcopenia)

Citation keys correspond to the bibliography.

##### 8.2.4.1 Research perspective

To overcome the barriers identified in Table 10, we posit that future research must shift toward constructing **Pathological Digital Phenotypes**. By utilizing physics engines (e.g., MuJoCo) for **Counterfactual Synthesis**, researchers can simulate pathological states with varying parameters (e.g., joint stiffness). This *Sim-to-Real* strategy, when constrained by established biomechanical rules (134) and augmented by generative techniques to produce realistic synthetic postures (135), can help ensure that synthetic data remains anatomically valid while providing the high-volume knowledge required for foundation model training.

#### 8.2.5 Clinical safety and ethics: biomechanical firewall and embodied trust

Given the scarcity of real-world data, Generative AI has become an essential tool for data augmentation and personalized feedback. However, this introduces the risk of **“Medical Hallucinations.”** If Generative Visual Biofeedback (VSM) deviates from physiological constraints (136), it may inadvertently induce patients to attempt anatomically impossible or hazardous movements.

##### 8.2.5.1 Ethical guardrails

Based on the security analysis of current technologies, we propose the implementation of a **“Biomechanical Firewall.”** This involves reverse-embedding physiological constraints (e.g., $E_{anat},E_{\;phys}$) into the generative loop as **Runtime Filters**. Any AI-generated instruction or video violating a patient’s personalized Range of Motion (ROM) must be intercepted and reverted to a safe state. Critically, this mechanism functions within a **Human-in-the-loop (HITL)** workflow: when the system detects high-risk compensatory patterns that exceed safety thresholds, it triggers an immediate alert for **human clinical review**, ensuring that AI-generated feedback remains under the supervision of qualified physiotherapists. Furthermore, leveraging the **Chain-of-Thought (CoT)** reasoning of MLLMs to provide anatomically consistent explanations (37) can make the complex features of AI more transparent. This causal interpretability serves as a logical cornerstone for establishing **Embodied Trust** among patients, clinicians, and AI agents, representing a key milestone for SaMD certification (97).

### 8.3 Limitations

This review has several limitations that should be considered when interpreting its findings. First, although the review followed the PRISMA 2020 reporting framework and was conducted according to a predefined search, screening, extraction, and evidence-mapping plan, it was not prospectively registered in PROSPERO. This limits the extent to which the review protocol can be independently verified. In addition, formal inter-rater reliability statistics, such as Cohen’s kappa, were not prospectively recorded during the original screening and eligibility phases. Although two reviewers independently re-checked the screening decisions, full-text eligibility decisions, and data extraction records during revision, the lack of prospectively recorded agreement statistics remains a methodological limitation.

The evidence base summarized in this review may also be affected by publication bias. Studies reporting successful computer vision-based rehabilitation systems, positive validation results, or promising prototype performance are more likely to appear in the published literature than studies reporting failed clinical validation, poor usability, low adherence, or negative implementation outcomes. Therefore, the current literature may present an overly optimistic view of the maturity and clinical readiness of vision-based rehabilitation technologies.

Another important limitation is the substantial heterogeneity of the included publications. The reviewed corpus included peer-reviewed clinical studies, engineering benchmarks, conference papers, systematic or scoping reviews, rehabilitation prototypes, protocols, and selected frontier preprints. The included populations ranged from stroke, Parkinson’s disease, osteoarthritis, sarcopenia, and orthopedic rehabilitation patients to healthy participants performing simulated rehabilitation tasks. Similarly, the reported outcomes varied widely, including MPJPE, joint angle error, ICC, RMSE, action quality scores, therapist ratings, usability, adherence, and selected clinical indicators. Because of this methodological, clinical, and outcome-level heterogeneity, a formal meta-analysis was not performed. Instead, this review used PAC-based evidence mapping and methodological appraisal to provide a structured qualitative synthesis.

The search strategy primarily focused on English-language literature and major international databases. Relevant studies published in Chinese, Japanese, Korean, German, or other languages may therefore have been underrepresented. This may introduce language-related selection bias, particularly because computer vision-based rehabilitation research is also active in non-English-speaking regions.

The rapidly evolving nature of Generative AI, MLLMs, and foundation-model-assisted rehabilitation represents another limitation. Selected frontier studies and preprints were included to contextualize emerging directions, but these records were treated as emerging evidence and were not weighted as equivalent to peer-reviewed clinical validation studies. Accordingly, recommendations related to MLLMs, autonomous rehabilitation agents, and generative visual feedback should be interpreted cautiously and updated as new peer-reviewed evidence becomes available.

Patient-reported outcomes were underrepresented in the reviewed literature. Many studies emphasized algorithmic or biomechanical metrics, such as pose-estimation error, ROM, ICC, RMSE, or action quality scores, whereas fewer studies systematically reported pain, perceived exertion, satisfaction, confidence, quality of life, functional independence, or long-term adherence. This limits the ability to determine whether technically accurate systems translate into meaningful patient-centered rehabilitation benefits. Future studies should integrate patient-reported outcomes alongside biomechanical, clinical, and usability endpoints.

Finally, population coverage remains incomplete. Although this review covered neurological, orthopedic, and geriatric rehabilitation populations, pediatric rehabilitation and populations with cognitive impairments, such as dementia or traumatic brain injury, were less extensively represented. These populations may require different feedback timing, interface design, caregiver involvement, safety monitoring, and motivational strategies. Future work should therefore validate vision-based rehabilitation systems across broader patient groups and real-world clinical contexts.

## 9 Conclusion

This paper has systematically reviewed the evolution of computer vision in rehabilitation training, establishing a structured taxonomic perspective through the unified “**Perception, Assessment, and Coaching (PAC)**” framework. Our analysis indicates a clear technological trajectory: (1) In the **Perception Domain**, the focus has shifted from simple geometric reconstruction to **Biomechanical Digital Twin** that ensure anatomical validity via physical constraints; (2) In the **Assessment Domain**, the research paradigm is evolving from empirical data fitting toward **Interpretable Pathological Attribution**; (3) In the **Coaching Domain**, feedback mechanisms are transitioning from rudimentary error reporting to an adaptive, multimodal **Sensorimotor Loop** designed to support motor relearning based on motor learning principles.

Notably, the deep penetration of **Generative AI** and **MLLMs** is fundamentally reconstructing these Domains. From intent-driven semantic sensing and CoT-based clinical reasoning to VSM-based generative feedback, large models are driving the evolution of rehabilitation systems from assistive tools into **Autonomous Rehab Agents** possessed of clinical logic. Although challenges such as pathological data distortion and clinical fidelity validation remain, the technical pathways established in this framework provide a clear roadmap for addressing the global shortage of rehabilitation resources and the deficit in rehabilitation literacy. Ultimately, the convergence of computer vision and rehabilitation medicine serves as a pivotal driver for the democratization of precision healthcare, providing a vital response to the challenges of a global aging society.

## Data Availability

As this is a review article, no primary datasets were generated. Existing datasets referenced in the literature are subject to their original licensing/access restrictions (e.g., some require institutional login, others prohibit commercial reuse), which are detailed in the respective source publications. Requests to access these datasets should be directed to Ping Ye, 20234080203@stu.usc.edu.cn.
