# Supplementary material for: Perception, assessment, and coaching: a systematic review and taxonomy of computer vision-based physical rehabilitation techniques
Source: Front Rehabil Sci. 2026 Jul 20;7:1906327. doi: 10.3389/fresc.2026.1906327 (PMC13429716; doi:10.3389/fresc.2026.1906327)
Supplement: Supplementary file 1 [file Datasheet1.pdf]

Supplementary Data Sheet 1

Database-specific search strategies and methodological quality appraisal

Manuscript: Perception, assessment, and coaching: A systematic review and taxonomy of computer vision-based physical rehabilitation techniques

Purpose. This supplementary data sheet supports the revised Methods section by providing (i) database-specific search strategies and (ii) a structured methodological quality appraisal/evidence weighting of the 147 cited records used in the PAC taxonomy and systematic review. The appraisal was used to contextualize evidence strength and was not used as an exclusion criterion.

Scope note. The complete author-supplied reference list contains 147 cited records. All 147 records are represented in Supplementary Table S2. To avoid over-weighting preliminary or contextual literature, each record is explicitly labeled by use in review, evidence status, and overall appraisal. Contextual/background studies, reviews, preprints, protocols, and benchmark-only studies were not weighted as equivalent to patient-level clinical validation evidence.

Supplementary Table S1. Database-specific search strategies

Note. All searches covered January 2010 to January 2026 unless otherwise stated. Because records from individual databases were deduplicated and consolidated before PRISMA screening, the main manuscript reports the combined database search count (n = 427) and additional snowballing/frontier tracing records (n = 31). Potential English-language selection bias is acknowledged in the manuscript Limitations.

Database / source	Search completion	Search period	Database-specific search string / syntax	Filters applied	Purpose in review
PubMed/MEDLINE	January 2026	Jan 2010-Jan 2026	("physical rehabilitation" OR "stroke rehabilitation" OR "orthopedic rehabilitation" OR "home-based rehabilitation" OR "telerehabilitation") AND ("computer vision" OR "human pose estimation" OR "markerless motion capture" OR "skeleton tracking" OR "3D pose estimation") AND ("movement assessment" OR "clinical validation" OR "biofeedback" OR "exercise feedback")	Humans where applicable; English-language publications; article/review filter where applicable; publication date 2010-2026	Clinical rehabilitation and medical evidence
Web of Science Core Collection	January 2026	Jan 2010-Jan 2026	TS=(("physical rehabilitation" OR "rehabilitation exercise" OR "telerehabilitation") AND ("computer vision" OR "human pose estimation" OR "markerless motion capture" OR "action quality assessment") AND ("movement assessment" OR "biofeedback" OR "coaching" OR "clinical validation"))	Article, review, proceeding paper; publication date 2010-2026; English where applicable	Multidisciplinary engineering, rehabilitation, and digital health literature
Scopus	January 2026	Jan 2010-Jan 2026	TITLE-ABS-KEY(("physical rehabilitation" OR "rehabilitation exercise" OR "telerehabilitation") AND ("computer vision" OR "human pose estimation" OR "skeleton tracking" OR "3D pose estimation" OR "action quality assessment") AND ("assessment" OR "coaching" OR "biofeedback" OR "exercise feedback"))	Article, conference paper, review; publication date 2010-2026; English where applicable	Broad engineering and clinical retrieval
IEEE Xplore	January 2026	Jan 2010-Jan 2026	("human pose estimation" OR "skeleton	Journals and conference proceedings;	Engineering systems, pose estimation,

Database / source	Search completion	Search period	Database-specific search string / syntax	Filters applied	Purpose in review
			tracking" OR "3D pose estimation" OR "action quality assessment" OR "computer vision") AND ("physical rehabilitation" OR "physiotherapy" OR "telerehabilitation" OR "exercise feedback")	publication date 2010-2026	and rehabilitation technology
ACM Digital Library	January 2026	Jan 2010-Jan 2026	("computer vision" OR "pose estimation" OR "skeleton tracking" OR "human-computer interaction") AND ("rehabilitation" OR "exercise feedback" OR "biofeedback" OR "coaching")	Research articles and proceedings; publication date 2010-2026	Human-computer interaction and interaction-system evidence
Cochrane Library	January 2026	Jan 2010-Jan 2026	("telerehabilitation" OR "physical rehabilitation" OR "rehabilitation exercise") AND ("video" OR "computer vision" OR "feedback" OR "biofeedback")	Trials and reviews where applicable; publication date 2010-2026	Clinical trial and review evidence
JMIR / JMIR family journals	January 2026	Jan 2010-Jan 2026	("digital rehabilitation" OR "telerehabilitation" OR "home-based rehabilitation") AND ("computer vision" OR "video-based assessment" OR "exercise feedback" OR "AI coaching")	JMIR digital health publications; publication date 2010-2026	Digital health and implementation-oriented studies
arXiv	January 2026	2024-2026 only	("generative AI" OR "multimodal large language model" OR "MLLM" OR "visual self-modeling" OR "foundation model") AND ("rehabilitation" OR "clinical reasoning" OR "movement assessment")	Preprints only; used for frontier technology discussion and not weighted as established clinical validation evidence	Frontier GenAI/MLLM and foundation-model context
Google Scholar / citation tracing	January 2026	Jan 2010-Jan 2026	Forward and backward citation tracing from core papers on markerless motion capture, rehabilitation assessment, action quality assessment, and feedback/coaching systems	Manual relevance screening against PICOS and PAC criteria	Snowballing and high-impact record identification

Supplementary Table S2. Conservative evidence classification and methodological quality appraisal of the 147 cited records

Appraisal framework. Each item was mapped according to PAC domain, use in review, rehabilitation population/context, study role, validation metrics or reference standards, real-world or external validation context, evidence status, and overall evidence appraisal. Categories support evidence weighting in the narrative synthesis and are not exclusion criteria. Full bibliographic titles are provided in the manuscript reference list; this table uses citation labels to keep the appraisal readable.

Overall appraisal categories. High = randomized/controlled clinical evidence or strong patient-level clinical validation; Moderate to high = patient-level or rehabilitation-specific validation with clinically relevant comparators/metrics; Moderate/technical = engineering, prototype, or benchmark validation; Secondary evidence = review/survey; Emerging = preprint, protocol, or frontier work; Contextual = background or rationale-supporting evidence. These categories were used for evidence weighting, not for excluding records from the review.

Coding note. "No ref. std. coded" or "No context coded" indicates conservative coding from extracted validation fields; it does not imply that the original article contained no validation information.

Ref ID	Citation	Publication type	PAC domain	Population / context	Study role / design	Reference standard / metrics	Validation context	Evidence status	Overall appraisal	Interpretive note	Use in review
ref1	Abedi et al., 2023	Conference proceeding	Perception / Assessment	Rehabilitation exercise	Technical / clinical validation	No ref. std. coded	No context coded	Peer-reviewed conference	Moderate / technical	Technical rehabilitation-exercise AQA evidence; useful for PAC mapping but not equivalent to clinical outcome validation.	Supporting technical evidence
ref2	Aguilar-Ortega et al., 2023	Journal article	Perception	Physical rehabilitation; Rehabilitation exercise	Dataset / technical benchmark	Pose benchmark; viewpoint/supine -pose evaluation	Benchmark / simulated rehabilitation setting	Peer-reviewed journal	Moderate / benchmark	Rehabilitation-relevant benchmark for viewpoint and posture domain shift; patient-level validation required.	Supporting benchmark evidence
ref3	Ahmed et al., 2021	Journal article	Assessment	Stroke / neurological rehabilitation	Technical / clinical validation	Automated movement assessment metrics	Clinical population indicated	Peer-reviewed journal	Moderate to high	Patient-level stroke rehabilitation assessment evidence; comparator and metric details should be interpreted from the study methods.	Core PAC evidence

Ref ID	Citation	Publication type	PAC domain	Population / context	Study role / design	Reference standard / metrics	Validation context	Evidence status	Overall appraisal	Interpretive note	Use in review
ref4	Antonj et al., 2025	Conference proceeding	Coaching	Pediatric rehabilitation; Home-based rehabilitation	System prototype / method	No ref. std. coded	Home / remote / mobile context indicated	Peer-reviewed conference	Moderate / technical	Home pediatric-adherence system tested in unimpaired participants; patient transferability should be interpreted cautiously.	Supporting technical evidence
ref5	Antunes et al., 2018	Conference proceeding	Perception	General computer vision / benchmark	Dataset / technical benchmark	ROM; 3D skeletal exercise-recognition metrics	Benchmark / simulated rehabilitation setting	Peer-reviewed conference	Moderate / benchmark	Technical benchmark evidence; not direct patient-level clinical validation.	Supporting benchmark evidence
ref6	Aoyagi et al., 2019	Journal article	Coaching	General / not specified	Motor-learning feedback intervention	No ref. std. coded	No context coded	Peer-reviewed journal	Contextual / motor-learning evidence	Peer-reviewed motor-learning feedback evidence; supports KR/KP discussion but is not a computer-vision rehabilitation validation study.	Motor-learning rationale
ref7	Averell et al., 2022	Journal article	Assessment	General / not specified	Contextual / supporting study	Compensatory movement detection	No context coded	Peer-reviewed journal	Moderate	Compensatory-movement detection evidence relevant to Assessment; clinical weighting depends on population and validation details.	Core PAC evidence
ref8	Aytekin et al., 2025	Conference proceeding	Perception	General computer vision / benchmark	Contextual / supporting study	ROM	No context coded	Peer-reviewed conference	Moderate / technical	Physics-based pose-estimation method supporting the Perception domain; not direct clinical validation evidence.	Supporting technical evidence
ref9	Bayle et al., 2023	Journal article	Assessment	Upper-limb rehabilitation; Healthy participants	Technical / clinical validation	Smoothness metrics; SPARC indicators	Upper-limb / healthy-participant metric validation	Peer-reviewed journal	Moderate / technical	Metric evidence for smoothness quantification; patient transferability should be cautious.	Supporting metric evidence

Ref ID	Citation	Publication type	PAC domain	Population / context	Study role / design	Reference standard / metrics	Validation context	Evidence status	Overall appraisal	Interpretive note	Use in review
ref10	Bazarevsky et al., 2020	Preprint	Perception	General / not specified	Supporting technical method	No ref. std. coded	No context coded	Preprint / emerging	Emerging	Preprint/frontier evidence; not weighted as established clinical validation; Technical benchmark/context; clinical generalization requires rehabilitation validation	Emerging/frontier
ref11	Bidulka et al., 2025	Journal article	Perception	General computer vision / benchmark	Contextual / supporting study	No ref. std. coded	No context coded	Peer-reviewed journal	Moderate / technical	Moderate/supporting evidence; interpret cautiously against clinical validation needs	Supporting technical evidence
ref12	Biebl et al., 2021	Journal article	Coaching	Knee / orthopedic rehabilitation; Hip / orthopedic rehabilitation	System prototype / method	No ref. std. coded	Clinical population indicated	Peer-reviewed journal	Moderate to high	Relevant rehabilitation validation; interpret according to comparator and sample characteristics	Core PAC evidence
ref13	Bruce et al., 2024	Journal article	Perception / Assessment	Rehabilitation exercise	Technical / clinical validation	No ref. std. coded	No context coded	Peer-reviewed journal	Moderate	Moderate/supporting evidence; interpret cautiously against clinical validation needs	Core PAC evidence
ref14	Cai et al., 2020	Journal article	Assessment	Stroke / neurological rehabilitation	Contextual / supporting study	compensatory movement	Clinical population indicated	Peer-reviewed journal	Moderate	Moderate/supporting evidence; interpret cautiously against clinical validation needs	Core PAC evidence
ref15	Capecci et al., 2019	Journal article	Perception / Assessment	Physical rehabilitation	Dataset / technical benchmark	KIMORE RGB-D dataset; clinical scores	Rehabilitation exercise dataset	Peer-reviewed journal	Moderate to high	Rehabilitation-specific dataset with clinical scores; useful for PAC mapping and assessment validation.	Supporting technical evidence
ref16	Chen et al., 2021	Journal article	Perception	General computer vision / benchmark	Contextual / supporting study	No ref. std. coded	No context coded	Peer-reviewed journal	Moderate / technical	Moderate/supporting evidence; interpret cautiously against clinical validation needs	Supporting technical evidence

Ref ID	Citation	Publication type	PAC domain	Population / context	Study role / design	Reference standard / metrics	Validation context	Evidence status	Overall appraisal	Interpretive note	Use in review
ref17	Chen et al., 2023	Journal article	PAC-related evidence	Older adults	Review / survey evidence	No ref. std. coded	No context coded	Secondary evidence	Secondary evidence	Secondary evidence; not weighted as primary validation	Secondary/contextual
ref18	Chen et al., 2020	Journal article	Perception	General computer vision / benchmark	Review / survey evidence	No ref. std. coded	No context coded	Secondary evidence	Secondary evidence	Secondary evidence; not weighted as primary validation; Technical benchmark/context; clinical generalization requires rehabilitation validation	Secondary/contextual
ref19	Cherian et al., 2021	Journal article	PAC-related evidence	General / not specified	Supporting technical method	No ref. std. coded	No context coded	Peer-reviewed journal	Technical benchmark/contextual	Technical benchmark/context; clinical generalization requires rehabilitation validation	Technical benchmark/context
ref20	Cui et al., 2020	Journal article	Context / background	Knee / orthopedic rehabilitation; Osteoarthritis / musculoskeletal	Contextual / background evidence	No ref. std. coded	Clinical population indicated	Peer-reviewed journal	Contextual	Contextual/background evidence; not part of primary quality weighting	Contextual/supporting
ref21	Cui et al., 2025	Journal article	PAC-related evidence	Hand / upper-limb function	Review / survey evidence	No ref. std. coded	No context coded	Secondary evidence	Secondary evidence	Secondary evidence; not weighted as primary validation; Technical benchmark/context; clinical generalization requires rehabilitation validation	Secondary/contextual
ref22	Cunha et al., 2025	Journal article	Perception	General computer vision / benchmark	Contextual / supporting study	No ref. std. coded	Home / remote / mobile context indicated	Peer-reviewed journal	Moderate	Moderate/supporting evidence; interpret cautiously against clinical validation needs	Core PAC evidence

Ref ID	Citation	Publication type	PAC domain	Population / context	Study role / design	Reference standard / metrics	Validation context	Evidence status	Overall appraisal	Interpretive note	Use in review
ref23	Deb et al., 2022	Journal article	Assessment	Physical rehabilitation; Rehabilitation exercise	Technical / clinical validation	No ref. std. coded	No context coded	Peer-reviewed journal	Moderate	Moderate/supporting evidence; interpret cautiously against clinical validation needs	Core PAC evidence
ref24	Desmarais et al., 2021	Journal article	Perception	General computer vision / benchmark	Review / survey evidence	No ref. std. coded	No context coded	Secondary evidence	Secondary evidence	Secondary evidence; not weighted as primary validation	Secondary/contextual
ref25	Di Mitri et al., 2022	Journal article	Coaching	General / not specified	Contextual / supporting study	No ref. std. coded	No context coded	Peer-reviewed journal	Moderate / technical	Moderate/supporting evidence; interpret cautiously against clinical validation needs	Supporting technical evidence
ref26	Dill et al., 2024	Journal article	Perception / Assessment	General / not specified	Technical / clinical validation	MPJPE / 3D reconstruction error	Controlled technical comparison	Peer-reviewed journal	Moderate / technical	Technical validation of reconstruction error; clinical interpretation requires reference-standard rehabilitation validation.	Supporting technical evidence
ref27	Dindorf et al., 2024	Journal article	Perception	General / not specified	Contextual / supporting study	No ref. std. coded	No context coded	Peer-reviewed journal	Moderate / technical	Moderate/supporting evidence; interpret cautiously against clinical validation needs	Supporting technical evidence
ref28	Dong et al., 2024	Journal article	Perception	General / not specified	Contextual / supporting study	No ref. std. coded	No context coded	Peer-reviewed journal	Moderate / technical	Moderate/supporting evidence; interpret cautiously against clinical validation needs	Supporting technical evidence
ref29	Falisse et al., 2020	Journal article	Perception	Musculoskeletal rehabilitation; Gait / mobility	Contextual / supporting study	IMU; gait parameters	No context coded	Peer-reviewed journal	Moderate / technical	Moderate/supporting evidence; interpret cautiously against clinical validation needs	Supporting technical evidence
ref30	Fang et al., 2022	Journal article	Perception	General / not specified	Supporting technical method	No ref. std. coded	No context coded	Peer-reviewed journal	Technical benchmark/contextual	Technical benchmark/context; clinical generalization requires rehabilitation validation	Technical benchmark/context

Ref ID	Citation	Publication type	PAC domain	Population / context	Study role / design	Reference standard / metrics	Validation context	Evidence status	Overall appraisal	Interpretive note	Use in review
ref31	Fang et al., 2022	Journal article	PAC-related evidence	General / not specified	Contextual / supporting study	No ref. std. coded	No context coded	Peer-reviewed journal	Moderate / technical	Moderate/supporting evidence; interpret cautiously against clinical validation needs	Supporting technical evidence
ref32	Feichtenhofer et al., 2019	Conference proceeding	Perception	General / not specified	Supporting technical method	No ref. std. coded	No context coded	Peer-reviewed conference	Technical benchmark/contextual	Technical benchmark/context; clinical generalization requires rehabilitation validation	Technical benchmark/context
ref33	Feigin et al., 2024	Journal article	Context / background	Stroke / neurological rehabilitation	Contextual / background evidence	No ref. std. coded	Clinical population indicated	Peer-reviewed journal	Contextual	Contextual/background evidence; not part of primary quality weighting	Contextual/supporting
ref34	Filtjens et al., 2022	Journal article	Perception	General / not specified	Contextual / supporting study	No ref. std. coded	No context coded	Peer-reviewed journal	Moderate / technical	Moderate/supporting evidence; interpret cautiously against clinical validation needs	Supporting technical evidence
ref35	Francesco et al., 2025	Journal article	PAC-related evidence	Hand / upper-limb function	Contextual / supporting study	No ref. std. coded	Clinical population indicated	Peer-reviewed journal	Moderate / technical	Moderate/supporting evidence; interpret cautiously against clinical validation needs	Supporting technical evidence
ref36	Gadhvi et al., 2025	Conference proceeding	PAC-related evidence	General / not specified	System prototype / method	No ref. std. coded	No context coded	Peer-reviewed conference	Moderate / technical	Moderate/supporting evidence; interpret cautiously against clinical validation needs	Supporting technical evidence
ref37	Galvan-Sosa et al., 2020	Journal article	PAC-related evidence	Healthcare / clinical context	Contextual / background evidence	ROM	Clinical population indicated	Peer-reviewed journal	Contextual	Contextual/background evidence; not part of primary quality weighting	Contextual/supporting
ref38	Gao et al., 2014	Conference proceeding	Perception / Assessment	General computer vision / benchmark	Dataset / technical benchmark	No ref. std. coded	Benchmark / simulated setting	Peer-reviewed conference	Technical benchmark/contextual	Technical benchmark/context; clinical generalization requires rehabilitation validation	Technical benchmark/context

Ref ID	Citation	Publication type	PAC domain	Population / context	Study role / design	Reference standard / metrics	Validation context	Evidence status	Overall appraisal	Interpretive note	Use in review
ref39	Gildea et al., 2022	Conference proceeding	Perception	General computer vision / benchmark	Contextual / supporting study	No ref. std. coded	No context coded	Peer-reviewed conference	Moderate / technical	Moderate/supporting evidence; interpret cautiously against clinical validation needs	Supporting technical evidence
ref40	Goldbraikh et al., 2024	Journal article	PAC-related evidence	General / not specified	Contextual / supporting study	No ref. std. coded	No context coded	Peer-reviewed journal	Moderate / technical	Moderate/supporting evidence; interpret cautiously against clinical validation needs	Supporting technical evidence
ref41	Graf et al., 2025	Conference proceeding	Perception	General / not specified	Contextual / supporting study	No ref. std. coded	No context coded	Peer-reviewed conference	Moderate / technical	Moderate/supporting evidence; interpret cautiously against clinical validation needs	Supporting technical evidence
ref42	Gupta et al., 2024	Conference proceeding	PAC-related evidence	General / not specified	Supporting technical method	No ref. std. coded	No context coded	Peer-reviewed conference	Technical benchmark/contextual	Technical benchmark/context; clinical generalization requires rehabilitation validation	Technical benchmark/context
ref43	Halilaj et al., 2018	Journal article	PAC-related evidence	General / not specified	Contextual / supporting study	No ref. std. coded	No context coded	Peer-reviewed journal	Moderate / technical	Moderate/supporting evidence; interpret cautiously against clinical validation needs	Supporting technical evidence
ref44	He et al., 2024	Journal article	Perception	Parkinson's disease / neurological rehabilitation; Gait / mobility	System prototype / method	Gait parameters; Parkinsonian gait features	Clinical Parkinson's disease population	Peer-reviewed journal	Moderate to high	Patient-level gait/mobility evidence for neurological rehabilitation; comparator details depend on original methods.	Core PAC evidence
ref45	He et al., 2024	Journal article	Perception	Older adults / sarcopenia; Older adults	Technical / clinical validation	TUG / mobility features; sarcopenia indicators	Clinical older-adult/sarcopenia population	Peer-reviewed journal	Moderate to high	Patient-level geriatric/mobility evidence; comparator details should be checked in original methods.	Core PAC evidence

Ref ID	Citation	Publication type	PAC domain	Population / context	Study role / design	Reference standard / metrics	Validation context	Evidence status	Overall appraisal	Interpretive note	Use in review
ref46	Hossain et al., 2023	Journal article	Assessment	Stroke / neurological rehabilitation; Upper-limb rehabilitation	Contextual / supporting study	No ref. std. coded	Clinical population indicated	Peer-reviewed journal	Moderate / technical	Moderate/supporting evidence; interpret cautiously against clinical validation needs	Supporting technical evidence
ref47	Hribernik et al., 2022	Journal article	Perception / Coaching	General / not specified	Review / survey evidence	No ref. std. coded	No context coded	Secondary evidence	Secondary evidence	Secondary evidence; not weighted as primary validation	Secondary/contextual
ref48	Hsu et al., 2024	Conference proceeding	Perception	General computer vision / benchmark	Contextual / supporting study	No ref. std. coded	No context coded	Peer-reviewed conference	Moderate / technical	Moderate/supporting evidence; interpret cautiously against clinical validation needs	Supporting technical evidence
ref49	Hu et al., 2023	Journal article	Perception / Assessment	Musculoskeletal rehabilitation	Technical / clinical validation	Vicon; ICC 0.84-0.98; angle error 5-10 deg	Clinical musculoskeletal gait assessment	Peer-reviewed journal	Moderate to high	Patient-level gait validation using Vicon; supports Perception/Assessment with setting-dependent interpretation.	Core PAC evidence
ref50	Hussain et al., 2024	Journal article	PAC-related evidence	Stroke / neurological rehabilitation	Contextual / supporting study	ROM; stroke/upper-limb movement indicators	Clinical stroke population	Peer-reviewed journal	Moderate	Supports clinical metric translation in Assessment; strength depends on validation design.	Core PAC evidence
ref51	Ionescu et al., 2013	Journal article	Perception	General computer vision / benchmark	Dataset / technical benchmark	No ref. std. coded	Benchmark / simulated setting	Peer-reviewed journal	Technical benchmark/contextual	Technical benchmark/context; clinical generalization requires rehabilitation validation	Technical benchmark/context
ref52	Ito et al., 2024	Journal article	Assessment	Stroke / neurological rehabilitation	Technical / clinical validation	Stroke movement-quality indicators	Clinical stroke rehabilitation population	Peer-reviewed journal	Moderate to high	Stroke assessment evidence relevant to pathological attribution; comparator details depend on study methods.	Core PAC evidence
ref53	Jesus et al., 2017	Journal article	Context / background	General / not specified	Contextual / background evidence	No ref. std. coded	No context coded	Peer-reviewed journal	Contextual	Contextual/background evidence; not part of primary quality weighting	Contextual/supporting

Ref ID	Citation	Publication type	PAC domain	Population / context	Study role / design	Reference standard / metrics	Validation context	Evidence status	Overall appraisal	Interpretive note	Use in review
ref54	Johansson et al., 2025	Journal article	Perception / Coaching	Stroke / neurological rehabilitation; Gait / mobility	Contextual / supporting study	ROM; gait parameters; stroke mobility indicators	Clinical stroke rehabilitation population	Peer-reviewed journal	Moderate to high	Stroke gait/mobility evidence supporting Perception/Coaching discussion; study-specific interpretation required.	Core PAC evidence
ref55	Kaku et al., 2022	Journal article	Perception	Stroke / neurological rehabilitation	Dataset / technical benchmark	No ref. std. coded	Benchmark / simulated setting	Peer-reviewed journal	Moderate	Moderate/supporting evidence; interpret cautiously against clinical validation needs	Core PAC evidence
ref56	Kay et al., 2017	Preprint	Perception	General computer vision / benchmark	Dataset / technical benchmark	No ref. std. coded	Benchmark / simulated setting	Preprint / emerging	Emerging	Preprint/frontier evidence; not weighted as established clinical validation; Technical benchmark/context; clinical generalization requires rehabilitation validation	Emerging/frontier
ref57	Kelly et al., 2019	Journal article	PAC-related evidence	Healthcare / clinical context	Contextual / background evidence	No ref. std. coded	Clinical population indicated	Peer-reviewed journal	Contextual	Contextual/background evidence; not part of primary quality weighting	Contextual/supporting
ref58	Kim et al., 2023	Journal article	Perception	General computer vision / benchmark	Supporting technical method	No ref. std. coded	No context coded	Peer-reviewed journal	Technical benchmark/contextual	Technical benchmark/context; clinical generalization requires rehabilitation validation	Technical benchmark/context
ref59	Knippenberg et al., 2017	Journal article	Perception	General / not specified	Review / survey evidence	No ref. std. coded	No context coded	Secondary evidence	Secondary evidence	Secondary evidence; not weighted as primary validation	Secondary/contextual
ref60	Koh et al., 2025	Journal article	Assessment	Stroke / neurological rehabilitation; Upper-limb rehabilitation	Contextual / supporting study	No ref. std. coded	Clinical population indicated	Peer-reviewed journal	Moderate	Moderate/supporting evidence; interpret cautiously against clinical validation needs	Core PAC evidence

Ref ID	Citation	Publication type	PAC domain	Population / context	Study role / design	Reference standard / metrics	Validation context	Evidence status	Overall appraisal	Interpretive note	Use in review
ref61	Koh et al., 2023	Journal article	PAC-related evidence	Stroke / neurological rehabilitation	Contextual / supporting study	No ref. std. coded	Clinical population indicated	Peer-reviewed journal	Moderate	Moderate/supporting evidence; interpret cautiously against clinical validation needs	Core PAC evidence
ref62	Kolarik et al., 2023	Journal article	Perception	Healthcare / clinical context	Review / survey evidence	No ref. std. coded	No context coded	Secondary evidence	Secondary evidence	Secondary evidence; not weighted as primary validation; Contextual/background evidence; not part of primary quality weighting	Contextual/supporting
ref63	Kryeem et al., 2023	Conference proceeding	Coaching	Hip / orthopedic rehabilitation; Home-based rehabilitation	System prototype / method	No ref. std. coded	Home / remote / mobile context indicated	Peer-reviewed conference	Moderate / technical	Moderate/supporting evidence; interpret cautiously against clinical validation needs	Core PAC evidence
ref64	Kuang et al., 2024	Journal article	Assessment	Rehabilitation exercise	Technical / clinical validation	No ref. std. coded	No context coded	Peer-reviewed journal	Moderate	Moderate/supporting evidence; interpret cautiously against clinical validation needs	Core PAC evidence
ref65	Lam et al., 2023	Journal article	Perception	Healthcare / clinical context	Review / survey evidence	No ref. std. coded	Clinical population indicated	Secondary evidence	Secondary evidence	Secondary evidence; not weighted as primary validation	Secondary/contextual
ref66	Lee et al., 2024	Journal article	PAC-related evidence	Stroke / neurological rehabilitation	Contextual / supporting study	No ref. std. coded	Clinical population indicated	Peer-reviewed journal	Moderate	Moderate/supporting evidence; interpret cautiously against clinical validation needs	Core PAC evidence
ref67	Lee et al., 2024	Journal article	Assessment / Coaching	Stroke / neurological rehabilitation	Contextual / supporting study	Compensatory movement detection	Clinical stroke rehabilitation population	Peer-reviewed journal	Moderate to high	Patient-level evidence for compensatory-pattern interpretation; supports Assessment/Coaching linkage.	Core PAC evidence

Ref ID	Citation	Publication type	PAC domain	Population / context	Study role / design	Reference standard / metrics	Validation context	Evidence status	Overall appraisal	Interpretive note	Use in review
ref68	Li et al., 2022	Conference proceeding	Perception	General computer vision / benchmark	Supporting technical method	No ref. std. coded	No context coded	Peer-reviewed conference	Technical benchmark/contextual	Technical benchmark/context; clinical generalization requires rehabilitation validation	Technical benchmark/context
ref69	Li et al., 2022	Journal article	Perception	General / not specified	Contextual / supporting study	ROM	No context coded	Peer-reviewed journal	Moderate / technical	Moderate/supporting evidence; interpret cautiously against clinical validation needs	Supporting technical evidence
ref70	Liang et al., 2025	Preprint	Perception	General / not specified	Emerging technical work	No ref. std. coded	No context coded	Preprint / emerging	Emerging	Preprint/frontier evidence; not weighted as established clinical validation	Emerging/frontier
ref71	Lin et al., 2014	Conference proceeding	Context / background	General / not specified	Dataset / technical benchmark	No ref. std. coded	No context coded	Peer-reviewed conference	Technical benchmark/contextual	Technical benchmark/context; clinical generalization requires rehabilitation validation	Technical benchmark/context
ref72	Liu et al., 2019	Journal article	Perception	General / not specified	Dataset / technical benchmark	No ref. std. coded	Benchmark / simulated setting	Peer-reviewed journal	Technical benchmark/contextual	Technical benchmark/context; clinical generalization requires rehabilitation validation	Technical benchmark/context
ref73	Lu et al., 2023	Journal article	PAC-related evidence	General / not specified	Review / survey evidence	No ref. std. coded	No context coded	Secondary evidence	Secondary evidence	Secondary evidence; not weighted as primary validation; Contextual/background evidence; not part of primary quality weighting	Contextual/supporting
ref74	Luciani et al., 2025	Journal article	Coaching	Upper-limb rehabilitation	Contextual / supporting study	No ref. std. coded	No context coded	Peer-reviewed journal	Moderate	Moderate/supporting evidence; interpret cautiously against clinical validation needs	Core PAC evidence

Ref ID	Citation	Publication type	PAC domain	Population / context	Study role / design	Reference standard / metrics	Validation context	Evidence status	Overall appraisal	Interpretive note	Use in review
ref75	Lugaresi et al., 2019	Preprint	Perception	General / not specified	Supporting technical method	No ref. std. coded	No context coded	Preprint / emerging	Emerging	Preprint/frontier evidence; not weighted as established clinical validation; Technical benchmark/context; clinical generalization requires rehabilitation validation	Emerging/frontier
ref76	Mahmood et al., 2019	Conference proceeding	Perception	General / not specified	Dataset / technical benchmark	No ref. std. coded	No context coded	Peer-reviewed conference	Technical benchmark/contextual	Technical benchmark/context; clinical generalization requires rehabilitation validation	Technical benchmark/context

Ref ID	Citation	Publication type	PAC domain	Population / context	Study role / design	Reference standard / metrics	Validation context	Evidence status	Overall appraisal	Interpretive note	Use in review
ref77	Martnez et al., 2019	Journal article	Perception	General / not specified	Contextual / supporting study	No ref. std. coded	No context coded	Thesis / dissertation	Moderate / technical	Moderate/supporting evidence; interpret cautiously against clinical validation needs	Supporting technical evidence
ref78	Marusic et al., 2025	Conference proceeding	Perception / Assessment / Coaching	Low back pain rehabilitation; Physical rehabilitation	Contextual / supporting study	No ref. std. coded	No context coded	Peer-reviewed conference	Moderate / technical	Moderate/supporting evidence; interpret cautiously against clinical validation needs	Core PAC evidence
ref79	Mehta et al., 2017	Conference proceeding	Perception	General computer vision / benchmark	Supporting technical method	No ref. std. coded	No context coded	Peer-reviewed conference	Technical benchmark/contextual	Technical benchmark/context; clinical generalization requires rehabilitation validation	Technical benchmark/context
ref80	Mesquita et al., 2025	Preprint	Perception / Assessment	Stroke / neurological rehabilitation; Rehabilitation exercise	Technical / clinical validation	ROM	Clinical population indicated	Preprint / emerging	Emerging	Preprint/frontier evidence; not weighted as established clinical validation	Emerging/frontier
ref81	Miao et al., 2024	Journal article	PAC-related evidence	Upper-limb rehabilitation	System prototype / method	No ref. std. coded	No context coded	Peer-reviewed journal	Moderate	Moderate/supporting evidence; interpret cautiously against clinical validation needs	Core PAC evidence
ref82	Milosevic et al., 2020	Journal article	Perception	Home-based rehabilitation	Contextual / supporting study	IMU; Kinect/RGB-D comparison	Home-based rehabilitation validation	Peer-reviewed journal	Moderate	Home rehabilitation comparator evidence using wearable and depth-camera modalities.	Core PAC evidence
ref83	Moccia et al., 2024	Journal article	PAC-related evidence	General / not specified	Contextual / background evidence	No ref. std. coded	No context coded	Peer-reviewed journal	Contextual	Contextual/background evidence; not part of primary quality weighting	Contextual/supporting
ref84	Naqvi et al., 2024	Journal article	Coaching	General / not specified	Contextual / supporting study	No ref. std. coded	Home / remote / mobile context indicated	Peer-reviewed journal	Moderate	Moderate/supporting evidence; interpret cautiously against clinical validation needs	Core PAC evidence

Ref ID	Citation	Publication type	PAC domain	Population / context	Study role / design	Reference standard / metrics	Validation context	Evidence status	Overall appraisal	Interpretive note	Use in review
ref85	Nikolaev et al., 2022	Journal article	Coaching	Stroke / neurological rehabilitation	Review / survey evidence	No ref. std. coded	Home / remote / mobile context indicated	Secondary evidence	Secondary evidence	Secondary evidence; not weighted as primary validation	Secondary/contextual
ref86	Osawa et al., 2023	Journal article	Perception / Coaching	General / not specified	System prototype / method	No ref. std. coded	Home / remote / mobile context indicated	Peer-reviewed journal	Moderate	Moderate/supporting evidence; interpret cautiously against clinical validation needs	Core PAC evidence
ref87	Owaki et al., 2021	Journal article	Coaching	Stroke / neurological rehabilitation	Contextual / supporting study	No ref. std. coded	Clinical population indicated	Peer-reviewed journal	Moderate	Moderate/supporting evidence; interpret cautiously against clinical validation needs	Core PAC evidence
ref88	Park et al., 2022	Journal article	PAC-related evidence	General / not specified	Contextual / supporting study	No ref. std. coded	No context coded	Peer-reviewed journal	Moderate / technical	Moderate/supporting evidence; interpret cautiously against clinical validation needs	Supporting technical evidence
ref89	Parmar et al., 2019	Conference proceeding	Assessment	General / not specified	Technical / clinical validation	AQA score	No context coded	Peer-reviewed conference	Moderate / technical	Moderate/supporting evidence; interpret cautiously against clinical validation needs	Core PAC evidence
ref90	Pavlakos et al., 2019	Conference proceeding	PAC-related evidence	Hand / upper-limb function	Supporting technical method	ROM	No context coded	Peer-reviewed conference	Technical benchmark/contextual	Technical benchmark/context; clinical generalization requires rehabilitation validation	Technical benchmark/context
ref91	Pavllo et al., 2019	Conference proceeding	Perception	General computer vision / benchmark	Contextual / supporting study	No ref. std. coded	No context coded	Peer-reviewed conference	Moderate / technical	Moderate/supporting evidence; interpret cautiously against clinical validation needs	Supporting technical evidence
ref92	Peng et al., 2023	Conference proceeding	PAC-related evidence	General / not specified	Contextual / supporting study	No ref. std. coded	No context coded	Peer-reviewed conference	Moderate / technical	Moderate/supporting evidence; interpret cautiously against clinical validation needs	Supporting technical evidence

Ref ID	Citation	Publication type	PAC domain	Population / context	Study role / design	Reference standard / metrics	Validation context	Evidence status	Overall appraisal	Interpretive note	Use in review
ref93	Pereira et al., 2023	Journal article	PAC-related evidence	Home-based rehabilitation	System prototype / method	No ref. std. coded	Home / remote / mobile context indicated	Peer-reviewed journal	Moderate	Moderate/supporting evidence; interpret cautiously against clinical validation needs	Core PAC evidence
ref94	Phanse et al., 2022	Journal article	PAC-related evidence	Healthcare / clinical context	Contextual / background evidence	No ref. std. coded	No context coded	Peer-reviewed journal	Contextual	Contextual/background evidence; not part of primary quality weighting	Contextual/supporting
ref95	Pintea et al., 2018	Conference proceeding	Perception	Hand / upper-limb function	Contextual / supporting study	No ref. std. coded	No context coded	Peer-reviewed conference	Moderate / technical	Moderate/supporting evidence; interpret cautiously against clinical validation needs	Supporting technical evidence
ref96	Pornpipatsakul et al., 2023	Conference proceeding	Perception	Stroke / neurological rehabilitation	System prototype / method	No ref. std. coded	Clinical population indicated	Peer-reviewed conference	Moderate / technical	Moderate/supporting evidence; interpret cautiously against clinical validation needs	Core PAC evidence
ref97	Qiao et al., 2024	Journal article	Perception	General / not specified	Contextual / supporting study	ROM	No context coded	Peer-reviewed journal	Moderate / technical	Moderate/supporting evidence; interpret cautiously against clinical validation needs	Supporting technical evidence
ref98	Rahman et al., 2026	Journal article	PAC-related evidence	General computer vision / benchmark	Supporting technical method	No ref. std. coded	No context coded	Peer-reviewed journal	Technical benchmark/contextual	Technical benchmark/context; clinical generalization requires rehabilitation validation	Technical benchmark/context
ref99	Reddy et al., 2025	Journal article	Perception / Assessment	Gait / mobility	Contextual / supporting study	IMU; gait parameters; temporal mobility features	Home/remote/mobile context	Peer-reviewed journal	Moderate	Wearable-comparator evidence relevant to remote gait assessment; clinical thresholds require caution.	Core PAC evidence

Ref ID	Citation	Publication type	PAC domain	Population / context	Study role / design	Reference standard / metrics	Validation context	Evidence status	Overall appraisal	Interpretive note	Use in review
ref100	Reiss et al., 2022	Preprint	Perception	General / not specified	Supporting technical method	No ref. std. coded	No context coded	Preprint / emerging	Emerging	Preprint/frontier evidence; not weighted as established clinical validation; Technical benchmark/context; clinical generalization requires rehabilitation validation	Emerging/frontier
ref101	Ren et al., 2024	Conference proceeding	Perception	General / not specified	Supporting technical method	No ref. std. coded	No context coded	Peer-reviewed conference	Technical benchmark/contextual	Technical benchmark/context; clinical generalization requires rehabilitation validation	Technical benchmark/context
ref102	Roth et al., 2025	Journal article	Context / background	Healthcare / clinical context	Contextual / supporting study	No ref. std. coded	No context coded	Peer-reviewed journal	Moderate / technical	Moderate/supporting evidence; interpret cautiously against clinical validation needs	Supporting technical evidence
ref103	Sadée et al., 2025	Journal article	Perception	Healthcare / clinical context	Contextual / background evidence	No ref. std. coded	No context coded	Peer-reviewed journal	Contextual	Contextual/background evidence; not part of primary quality weighting	Contextual/supporting
ref104	Saha et al., 2026	Journal article	Assessment / Coaching	Healthcare / clinical context	Technical / clinical validation	No ref. std. coded	Benchmark / simulated setting	Peer-reviewed journal	Emerging / technical	Emerging AI direction; requires prospective clinical validation	Supporting technical evidence
ref105	Schaffert et al., 2019	Journal article	PAC-related evidence	Hip / orthopedic rehabilitation	Review / survey evidence	No ref. std. coded	No context coded	Secondary evidence	Secondary evidence	Secondary evidence; not weighted as primary validation	Secondary/contextual
ref106	Schneider et al., 2024	Journal article	Coaching	General / not specified	Review / survey evidence	No ref. std. coded	No context coded	Secondary evidence	Secondary evidence	Secondary evidence; not weighted as primary validation; Emerging AI direction; requires prospective clinical validation	Secondary/contextual

Ref ID	Citation	Publication type	PAC domain	Population / context	Study role / design	Reference standard / metrics	Validation context	Evidence status	Overall appraisal	Interpretive note	Use in review
ref107	Seredin et al., 2025	Journal article	Perception	General / not specified	Contextual / supporting study	No ref. std. coded	No context coded	Peer-reviewed journal	Moderate	Moderate/supporting evidence; interpret cautiously against clinical validation needs	Core PAC evidence
ref108	Sethi et al., 2022	Journal article	Perception	Gait / mobility	Review / survey evidence	gait parameters	No context coded	Secondary evidence	Secondary evidence	Secondary evidence; not weighted as primary validation	Secondary/contextual
ref109	Sherif et al., 2025	Preprint	Assessment	Rehabilitation exercise	Technical / clinical validation	No ref. std. coded	No context coded	Preprint / emerging	Emerging	Preprint/frontier evidence; not weighted as established clinical validation	Emerging/frontier
ref110	Shetty et al., 2023	Conference proceeding	Perception	General / not specified	Contextual / supporting study	No ref. std. coded	No context coded	Peer-reviewed conference	Moderate / technical	Moderate/supporting evidence; interpret cautiously against clinical validation needs	Supporting technical evidence
ref111	Shoitan et al., 2026	Journal article	PAC-related evidence	General / not specified	Review / survey evidence	No ref. std. coded	No context coded	Secondary evidence	Secondary evidence	Secondary evidence; not weighted as primary validation; Emerging AI direction; requires prospective clinical validation	Secondary/contextual
ref112	SIMOES et al., 2024	Journal article	Perception / Assessment	Physiotherapy	Supporting technical method	accuracy	No context coded	Peer-reviewed journal	Technical benchmark/contextual	Technical benchmark/context; clinical generalization requires rehabilitation validation	Core PAC evidence
ref113	Singhal et al., 2025	Journal article	Coaching	Healthcare / clinical context	Contextual / background evidence	No ref. std. coded	No context coded	Peer-reviewed journal	Contextual	Contextual/background evidence; not part of primary quality weighting; Emerging AI direction; requires prospective clinical validation	Contextual/supporting

Ref ID	Citation	Publication type	PAC domain	Population / context	Study role / design	Reference standard / metrics	Validation context	Evidence status	Overall appraisal	Interpretive note	Use in review
ref114	Song et al., 2024	Conference proceeding	Perception	General / not specified	Contextual / supporting study	No ref. std. coded	No context coded	Peer-reviewed conference	Moderate / technical	Moderate/supporting evidence; interpret cautiously against clinical validation needs	Supporting technical evidence

Ref ID	Citation	Publication type	PAC domain	Population / context	Study role / design	Reference standard / metrics	Validation context	Evidence status	Overall appraisal	Interpretive note	Use in review
ref115	Sonoda et al., 2024	Journal article	Assessment	General / not specified	Contextual / background evidence	No ref. std. coded	No context coded	Peer-reviewed journal	Contextual	Contextual/background evidence; not part of primary quality weighting	Contextual/supporting
ref116	Stenum et al., 2021	Journal article	Perception	Gait / mobility	Contextual / supporting study	IMU comparator; gait parameters	Controlled gait-analysis validation	Peer-reviewed journal	Moderate	Markerless gait-analysis evidence with clinically relevant comparator metrics.	Core PAC evidence
ref117	Sun et al., 2024	Conference proceeding	Assessment / Coaching	Rehabilitation exercise	Technical / clinical validation	AQA score; semantic corrective feedback	Technical rehabilitation-exercise validation	Peer-reviewed conference	Moderate / technical	Core Assessment/Coaching evidence for semantic translation; not RCT evidence for KP effectiveness.	Core PAC evidence
ref118	Tang et al., 2025	Conference proceeding	Assessment / Coaching	Rehabilitation exercise	Technical / clinical validation	ROM; LLM-based feedback/corrective cues	Technical validation; clinical validation pending	Peer-reviewed conference	Emerging / technical	Emerging LLM-assisted feedback evidence; not established clinical effectiveness evidence.	Core PAC evidence
ref119	Ukey et al., 2025	Journal article	Assessment	Stroke / neurological rehabilitation; Hand / upper-limb function	Technical / clinical validation	No ref. std. coded	Clinical population indicated	Peer-reviewed journal	Moderate	Moderate/supporting evidence; interpret cautiously against clinical validation needs	Core PAC evidence
ref120	Ullah et al., 2025	Conference proceeding	PAC-related evidence	Parkinson's disease / neurological rehabilitation	System prototype / method	No ref. std. coded	Clinical population indicated	Peer-reviewed conference	Moderate / technical	Moderate/supporting evidence; interpret cautiously against clinical validation needs	Core PAC evidence
ref121	Vakanski et al., 2018	Journal article	PAC-related evidence	Physical rehabilitation; Rehabilitation exercise	Dataset / technical benchmark	No ref. std. coded	No context coded	Peer-reviewed journal	Moderate	Moderate/supporting evidence; interpret cautiously against clinical validation needs	Core PAC evidence
ref122	Venkatesan et al., 2021	Journal article	Coaching	Healthcare / clinical context	Contextual / background evidence	No ref. std. coded	No context coded	Peer-reviewed journal	Contextual	Contextual/background evidence; not part of primary quality weighting	Contextual/supporting

Ref ID	Citation	Publication type	PAC domain	Population / context	Study role / design	Reference standard / metrics	Validation context	Evidence status	Overall appraisal	Interpretive note	Use in review
ref123	Vineeth et al., 2024	Conference proceeding	Assessment	Physiotherapy	Technical / clinical validation	No ref. std. coded	No context coded	Peer-reviewed conference	Moderate / technical	Moderate/supporting evidence; interpret cautiously against clinical validation needs	Core PAC evidence
ref124	Wang et al., 2025	Journal article	Context / background	Stroke / neurological rehabilitation	Contextual / background evidence	No ref. std. coded	Clinical population indicated	Peer-reviewed journal	Contextual	Contextual/background evidence; not part of primary quality weighting	Contextual/supporting
ref125	Wang et al., 2023	Journal article	Coaching	Meniscus injury / orthopedic rehabilitation	Protocol; outcomes not available	No ref. std. coded	No context coded	Peer-reviewed journal	Emerging / protocol	Protocol only; no outcome evidence available	Emerging/frontier
ref126	Willingham et al., 2024	Journal article	PAC-related evidence	General / not specified	Contextual / supporting study	No ref. std. coded	No context coded	Peer-reviewed journal	Moderate	Moderate/supporting evidence; interpret cautiously against clinical validation needs	Core PAC evidence
ref127	Woznowski et al., 2016	Book chapter / conference proceeding	Context / background	Healthcare / clinical context	Contextual / supporting study	No ref. std. coded	Home / remote / mobile context indicated	Incollection	Moderate / technical	Moderate/supporting evidence; interpret cautiously against clinical validation needs	Supporting technical evidence
ref128	Xu et al., 2023	Conference proceeding	Perception	General computer vision / benchmark	Contextual / supporting study	No ref. std. coded	No context coded	Peer-reviewed conference	Moderate / technical	Moderate/supporting evidence; interpret cautiously against clinical validation needs	Supporting technical evidence
ref129	Xu et al., 2025	Journal article	Assessment	General / not specified	Technical / clinical validation	AQA score	No context coded	Peer-reviewed journal	Moderate	Moderate/supporting evidence; interpret cautiously against clinical validation needs	Core PAC evidence
ref130	Xu et al., 2022	Journal article	Perception	General computer vision / benchmark	Supporting technical method	No ref. std. coded	No context coded	Peer-reviewed journal	Technical benchmark/contextual	Technical benchmark/context; clinical generalization requires rehabilitation validation	Technical benchmark/context

Ref ID	Citation	Publication type	PAC domain	Population / context	Study role / design	Reference standard / metrics	Validation context	Evidence status	Overall appraisal	Interpretive note	Use in review
ref131	Yang et al., 2023	Journal article	Assessment	General / not specified	Review / survey evidence	No ref. std. coded	No context coded	Secondary evidence	Secondary evidence	Secondary evidence; not weighted as primary validation; Technical benchmark/context; clinical generalization requires rehabilitation validation	Secondary/contextual
ref132	Yang et al., 2025	Conference proceeding	Perception	General / not specified	Supporting technical method	No ref. std. coded	No context coded	Peer-reviewed conference	Technical benchmark/contextual	Technical benchmark/context; clinical generalization requires rehabilitation validation	Technical benchmark/context
ref133	Yao et al., 2023	Journal article	Assessment	Physical rehabilitation; Rehabilitation exercise	Technical / clinical validation	No ref. std. coded	No context coded	Peer-reviewed journal	Moderate	Moderate/supporting evidence; interpret cautiously against clinical validation needs	Core PAC evidence
ref134	Yeung et al., 2021	Journal article	Coaching	General / not specified	Contextual / background evidence	No ref. std. coded	No context coded	Peer-reviewed journal	Contextual	Contextual/background evidence; not part of primary quality weighting	Contextual/supporting
ref135	Yeung et al., 2023	Preprint	Perception / Assessment / Coaching	Stroke / neurological rehabilitation; Upper-limb rehabilitation	Technical / clinical validation	Kinect comparator; therapist-guided rating	Home/remote stroke upper-limb context	Preprint / emerging	Emerging	Emerging SmartRehab evidence; clinically relevant but limited by preprint status.	Emerging/frontier
ref136	Yin et al., 2025	Preprint	Assessment	General / not specified	Technical / clinical validation	AQA score	No context coded	Preprint / emerging	Emerging / secondary	Preprint survey; secondary and frontier evidence, not primary clinical validation	Emerging/frontier
ref137	Yoshimura et al., 2024	Conference proceeding	Perception	General computer vision / benchmark	Dataset / technical benchmark	No ref. std. coded	Benchmark / simulated setting	Peer-reviewed conference	Technical benchmark/contextual	Technical benchmark/context; clinical generalization requires rehabilitation validation	Technical benchmark/context

Ref ID	Citation	Publication type	PAC domain	Population / context	Study role / design	Reference standard / metrics	Validation context	Evidence status	Overall appraisal	Interpretive note	Use in review
ref138	Yu et al., 2021	Conference proceeding	PAC-related evidence	General / not specified	Supporting technical method	No ref. std. coded	No context coded	Peer-reviewed conference	Technical benchmark/contextual	Technical benchmark/context; clinical generalization requires rehabilitation validation	Technical benchmark/context
ref139	Zhang et al., 2023	Journal article	Assessment	General / not specified	Technical / clinical validation	No ref. std. coded	No context coded	Peer-reviewed journal	Moderate	Moderate/supporting evidence; interpret cautiously against clinical validation needs	Core PAC evidence
ref140	Zhang et al., 2022	Journal article	Coaching	Musculoskeletal rehabilitation	Review / survey evidence	No ref. std. coded	Clinical population indicated	Secondary evidence	Secondary evidence	Secondary evidence; not weighted as primary validation	Secondary/contextual
ref141	Zhao et al., 2023	Journal article	Perception	General computer vision / benchmark	Supporting technical method	No ref. std. coded	No context coded	Peer-reviewed journal	Technical benchmark/contextual	Technical benchmark/context; clinical generalization requires rehabilitation validation	Technical benchmark/context
ref142	Zheng et al., 2023	Journal article	Perception	General computer vision / benchmark	Review / survey evidence	No ref. std. coded	No context coded	Secondary evidence	Secondary evidence	Secondary evidence; not weighted as primary validation; Technical benchmark/context; clinical generalization requires rehabilitation validation	Secondary/contextual
ref143	Zheng et al., 2021	Conference proceeding	Perception	General computer vision / benchmark	Supporting technical method	No ref. std. coded	No context coded	Peer-reviewed conference	Technical benchmark/contextual	Technical benchmark/context; clinical generalization requires rehabilitation validation	Technical benchmark/context
ref144	Zhou et al., 2024	Preprint	Assessment	General / not specified	Dataset / technical benchmark	AQA score	Benchmark / simulated setting	Preprint / emerging	Emerging / secondary	Preprint survey/benchmark; secondary and frontier evidence, not primary clinical validation	Emerging/frontier

Ref ID	Citation	Publication type	PAC domain	Population / context	Study role / design	Reference standard / metrics	Validation context	Evidence status	Overall appraisal	Interpretive note	Use in review
ref145	Zhu et al., 2025	Journal article	Coaching	Healthcare / clinical context	Contextual / background evidence	No ref. std. coded	No context coded	Peer-reviewed journal	Contextual	Contextual/background evidence; not part of primary quality weighting; Emerging AI direction; requires prospective clinical validation	Contextual/supporting
ref146	Zhu et al., 2023	Journal article	Perception / Assessment	Gait / mobility	Technical / clinical validation	Vicon comparison; gait scores/correlation	Controlled gait-analysis validation	Peer-reviewed journal	Moderate to high	Motion-capture comparison supports gait metric interpretation; task- and setting-dependent.	Core PAC evidence
ref147	Zhu et al., 2022	Conference proceeding	Perception	General computer vision / benchmark	Contextual / supporting study	No ref. std. coded	No context coded	Peer-reviewed conference	Moderate / technical	Moderate/supporting evidence; interpret cautiously against clinical validation needs	Supporting technical evidence
