# Supplementary material for: Perception, assessment, and coaching: a systematic review and taxonomy of computer vision-based physical rehabilitation techniques
Source: Front Rehabil Sci. 2026 Jul 20;7:1906327. doi: 10.3389/fresc.2026.1906327 (PMC13429716; doi:10.3389/fresc.2026.1906327)
Supplement: Supplementary file 3 [file Image1.pdf]

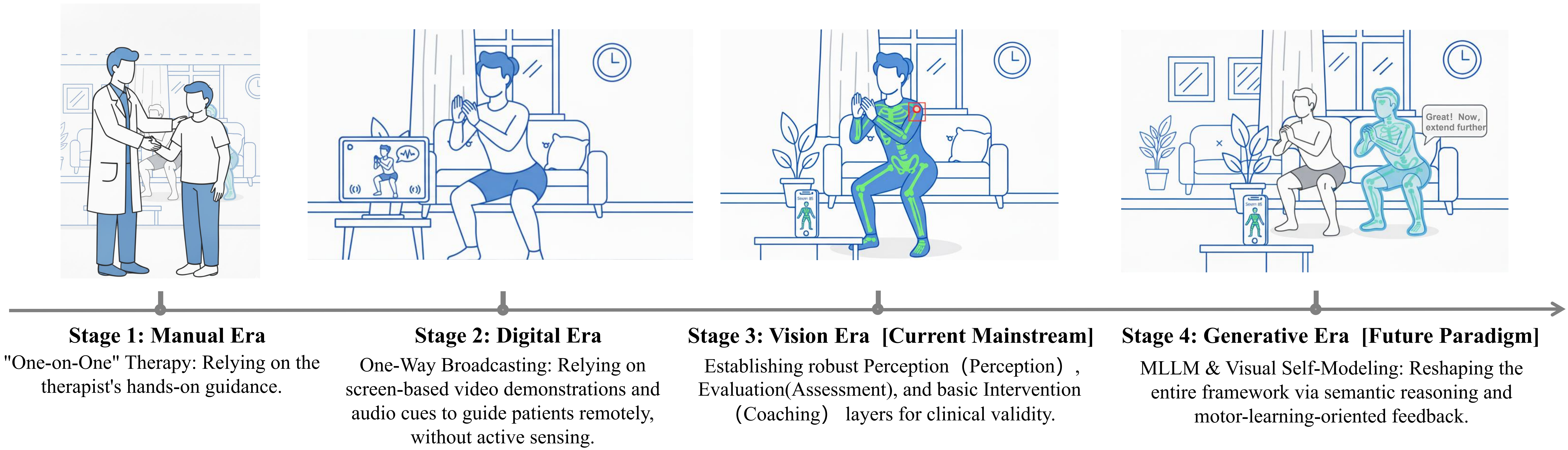

Great! Now, extend further
Stage 1: Manual Era
"One-on-One" Therapy: Relying on the therapist's hands-on guidance.
Stage 2: Digital Era
One-Way Broadcasting: Relying on screen-based video demonstrations and audio cues to guide patients remotely, without active sensing.
Stage 3: Vision Era [Current Mainstream]
Establishing robust Perception（Perception）, Evaluation(Assessment), and basic Intervention （Coaching） layers for clinical validity.
Stage 4: Generative Era [Future Paradigm]
MLLM & Visual Self-Modeling: Reshaping the entire framework via semantic reasoning and motor-learning-oriented feedback.
