# Supplementary figures and images for: Perception, assessment, and coaching: a systematic review and taxonomy of computer vision-based physical rehabilitation techniques
Source: Front Rehabil Sci. 2026 Jul 20;7:1906327. doi: 10.3389/fresc.2026.1906327 (PMC13429716; doi:10.3389/fresc.2026.1906327)

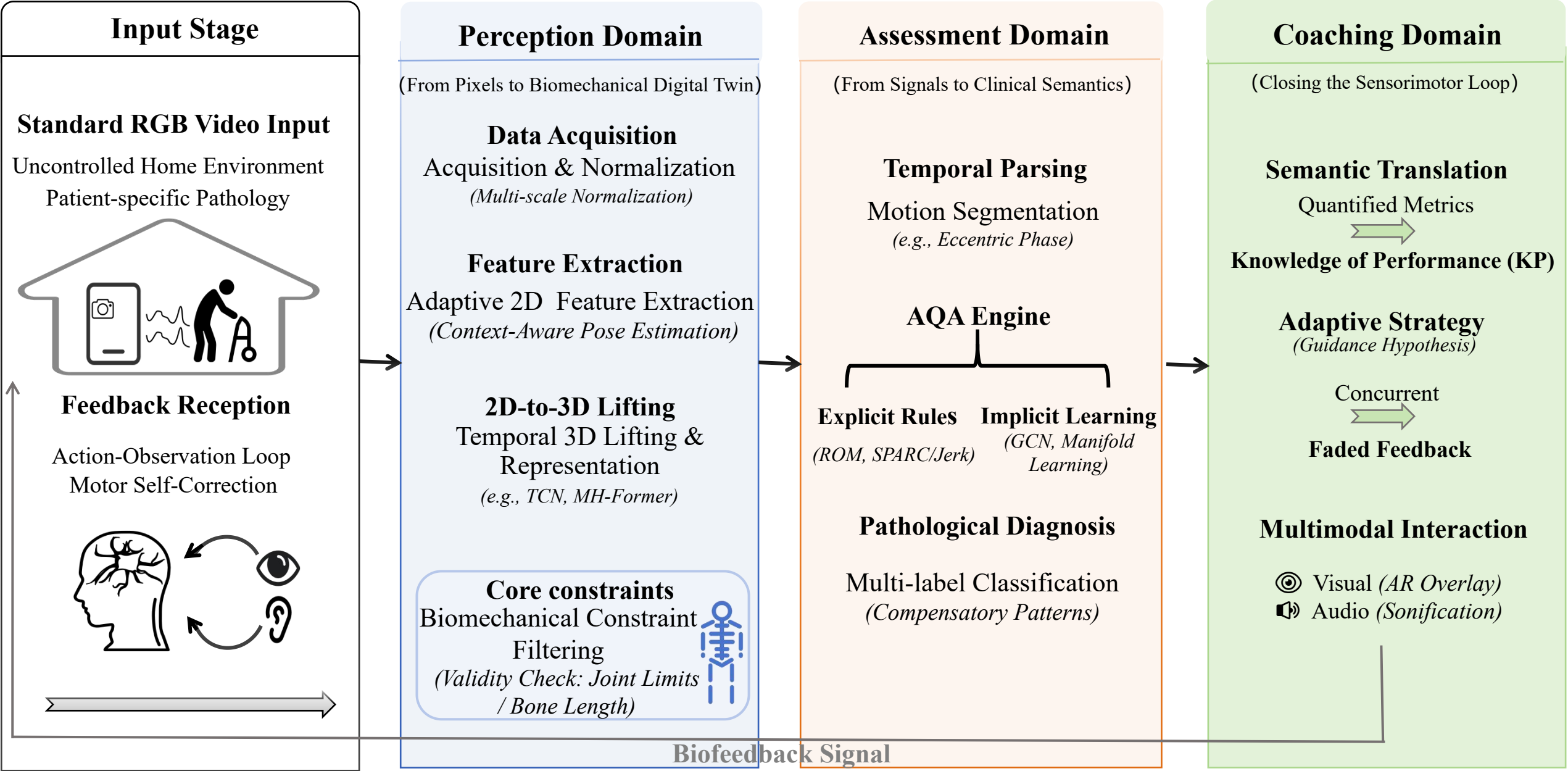

Supplement: Supplementary file 4 [file Image2.pdf]

HPE  
for Rehabilitation

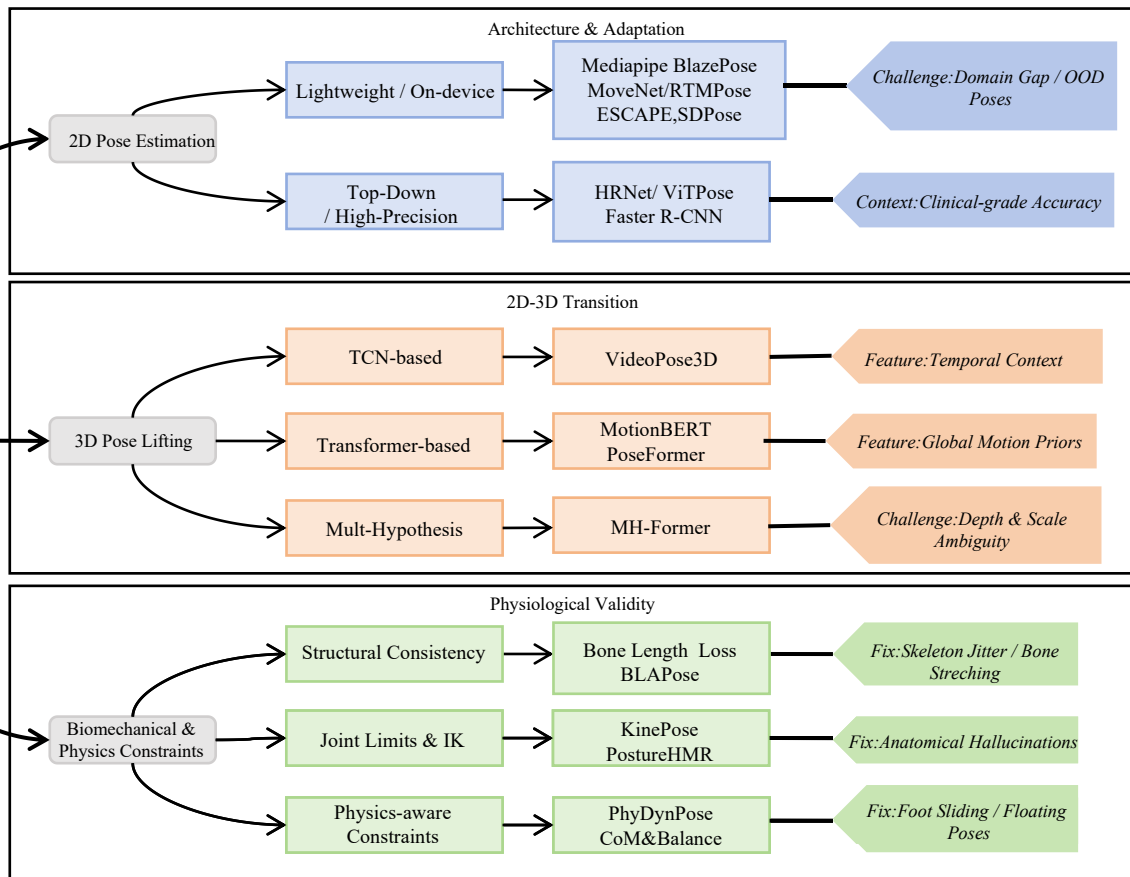

Supplement: Supplementary file 5 [file Image3.pdf]
